# Rapid identification of species, sex and maturity by mass spectrometric analysis of animal faeces

**DOI:** 10.1186/s12915-019-0686-9

**Published:** 2019-08-14

**Authors:** Nicola B. Davidson, Natalie I. Koch, Joscelyn Sarsby, Emrys Jones, Jane L. Hurst, Robert J. Beynon

**Affiliations:** 1 Mammalian Behaviour and Evolution Group, Institute of Integrative Biology, University of Liverpool, Leahurst Campus, Neston, CH64 7TE UK; 2 Centre for Proteome Research, Institute of Integrative Biology, University of Liverpool, Liverpool, L69 7ZB UK; 3 Waters Corporation, Stamford Avenue, Altrincham Road, Wilmslow, SK9 4AX UK

**Keywords:** Faecal analysis, Faeces, Mass spectrometry, Species identification, Rodents

## Abstract

**Background:**

We describe a new approach to the recovery of information from faecal samples, based on the analysis of the molecular signature generated by rapid evaporative ionisation mass spectrometry (REIMS).

**Results:**

Faecal pellets from five different rodent species were analysed by REIMS, and complex mass spectra were acquired rapidly (typically a few seconds per sample). The uninterpreted mass spectra (signatures) were then used to seed linear discriminant analysis and classification models based on random forests. It was possible to classify each species of origin with a high rate of accuracy, whether faeces were from animals maintained under standard laboratory conditions or wild-caught. REIMS signatures were stable to prior storage of the faecal material under a range of different conditions and were not altered rapidly or radically by changes in diet. Further, within species, REIMS signatures could be used to discriminate faeces from adult versus juvenile mice, male versus female mice and those from three different laboratory strains.

**Conclusions:**

REIMS offers a completely novel method for the rapid analysis of faecal samples, extending faecal analysis (previously focused on DNA) to an assessment of phenotype, and has considerable potential as a new tool in the armamentarium of the field biologist.

**Electronic supplementary material:**

The online version of this article (10.1186/s12915-019-0686-9) contains supplementary material, which is available to authorized users.

## Introduction

Faeces are a common ‘calling card’ left by animals in the wild, and such deposits have proven to be a valuable source of information regarding species [1, 2], sex [3], diet [4] and physiological status, notably stress hormone metabolites [5]. Identification of species in the field is required for a variety of reasons, including pest control, conservation and scientific research [1, 6, 7]. Collection of faeces is less labour intensive than methods to detect or capture live animals; furthermore, faeces may be the only material available to identify cryptic species [1, 8]. The predominant modes of faecal analysis are based on a visual categorisation of macroscopic dietary remains (such bones or wing carapaces) or by analysis of DNA, both of which can be protracted and require expert skills [9, 10]. There is a scope for novel approaches to faecal analysis to supplement and support such methods, especially for new methods that are rapid and easily applied.

One of the major areas of development in biological mass spectrometry has been the development of new ambient ion sources that permit mass spectral data to be collected without prior sample preparation [11–13]. The relatively new technique of rapid evaporative ionisation mass spectrometry (REIMS) provides a new potential method for the analysis of information contained in faeces. In REIMS acquisition, samples are subjected to a high-frequency alternating current that generates heat in the sample which, in turn, creates an aerosol containing biological molecules. The molecules are then subjected to ‘soft’ ionisation that generates information-rich molecular ions [14]. To date, REIMS has found applications in the provision of new information during surgical diathermy (electrosurgery) [11, 14–16], in the examination of foodstuffs primarily for the analysis of species of origin and adulteration and in microbial typing [12, 17, 18]. REIMS can be used with solid or semi-solid samples and requires little or no sample preparation or prior separation before analysis [11, 12].

We were intrigued by the possibility of using REIMS to analyse faecal material, providing a new molecular profile that would relate to phenotype, and thus supplement or provide an alternative to faecal DNA analysis. We particularly wanted to explore species identification using REIMS on rodent faecal samples due to the difficulty in distinguishing faecal pellets from similarly sized rodent species. We have completed an analysis of faecal samples from several rodent species, maintained under standard laboratory conditions and collected from the natural environment. We report that individual species are clearly resolvable with a high degree of confidence, that the signatures are robust to storage and that REIMS can be a powerful new tool in biological and environmental research. To explore the potential of distinguishing additional phenotypic information about individuals within a species, our analysis of samples from laboratory mice shows that faecal samples from adults can be discriminated from juveniles, that male faeces can be discriminated from female faeces and, further, that genetically distinct inbred strains can also be resolved.

## Results

### Faecal samples generate informative REIMS data

**Fig. 1:**
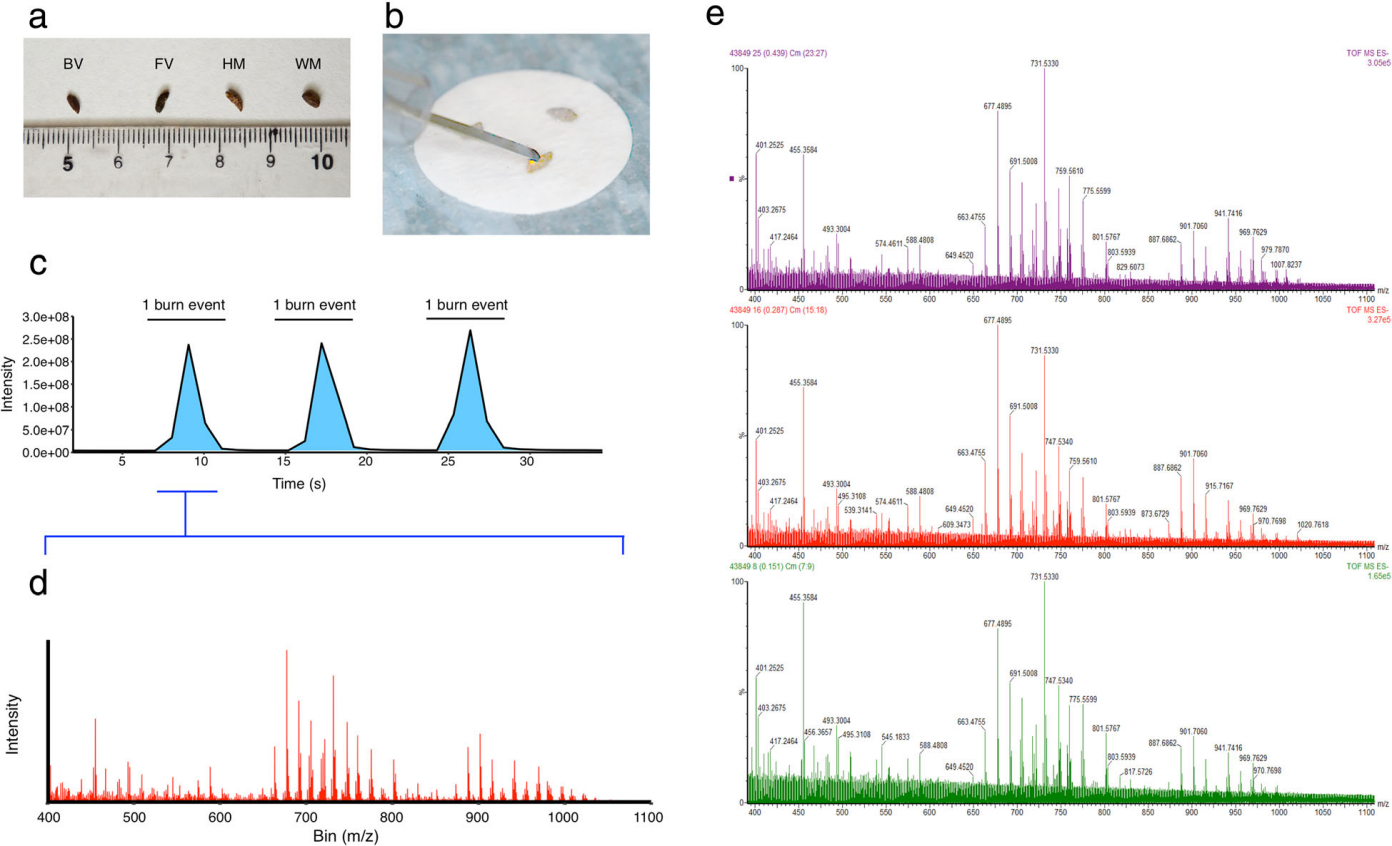
Overview of REIMS workflow. Faecal pellets from four small rodents (BV: bank vole, *Myodes glareolus*; FV: field vole, *Microtus agrestis*; HM: house mouse, *Mus musculus domesticus*; WM: wood mouse, *Apodemus sylvaticus*) are visually indistinguishable (**a**) (*Rattus norvegicus* not included as adult pellets are easily distinguishable by size, being much larger than the others). The faecal pellets are hydrated and burned using a monopolar diathermy electrode (**b**). Each burn event results in a burst of ion current at the mass spectrometer (**c**) resulting in a complex mass spectrum (**d**). Mass spectra of three discrete faecal pellets from one individual (wood mouse), which yielded very similar patterns of ions (**e**)z`

**Fig. 2:**
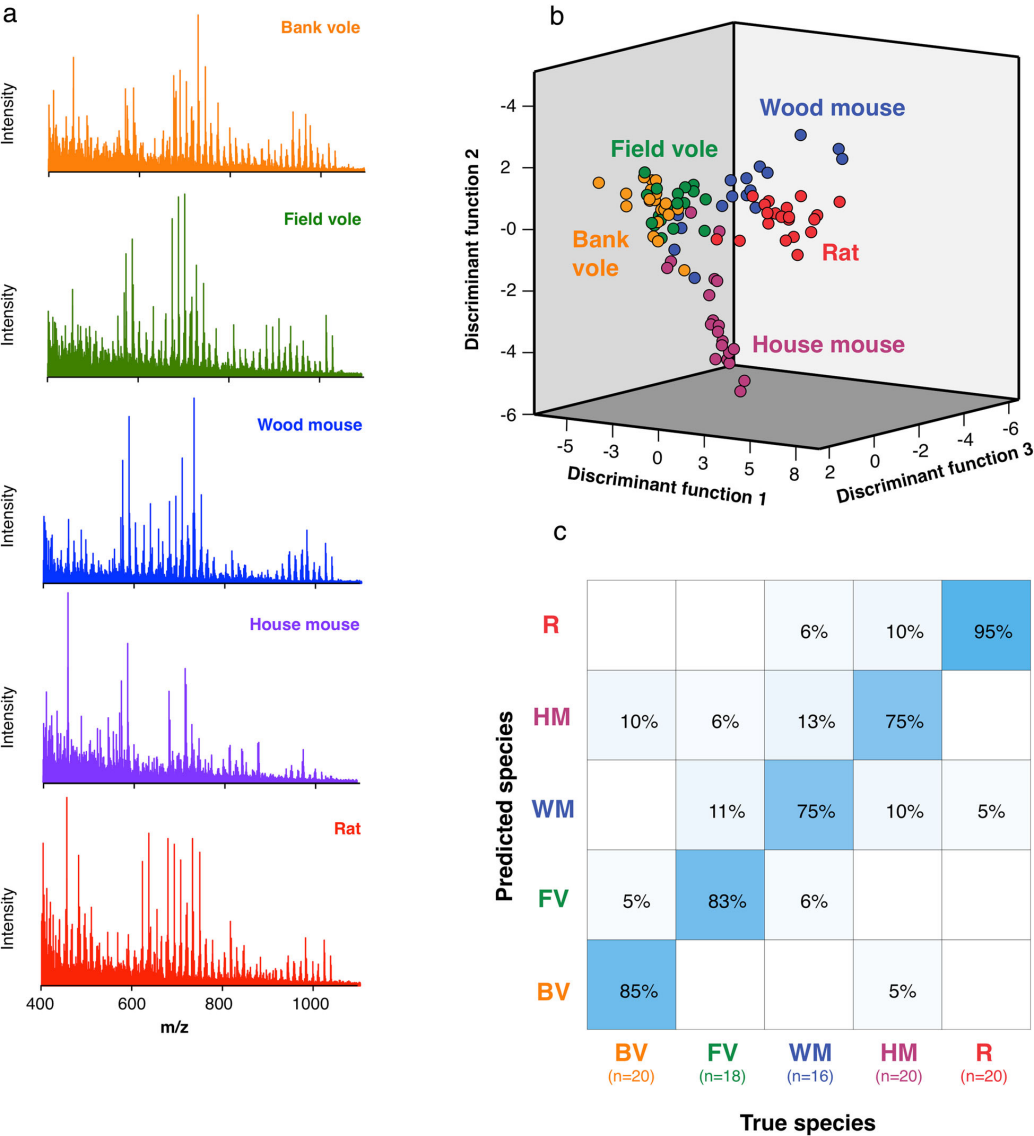
REIMS discrimination of faecal pellets from 5 rodent species under laboratory conditions. REIMS data were collected for faecal pellets from 16 to 20 individuals (including both sexes) for 5 different rodent species. All animals were fed the same base laboratory diet, but wood mice and voles also received supplements. Mass spectra were collected, binned and averaged per individual. **a** Spectra averaged across individuals of the 5 different species. **b** Discriminant function analysis of the top 12 principal components for classification of bank voles (BV, orange points), field voles (FV, green points), house mice (HM, purple points), wood mice (WM, blue points) and Wistar × Brown Norway rats (R, red points). **c** Random forest confusion matrix for species classification, using all data, expressed as the percentage of each species classified correctly or incorrectly (highlighted in blue)

A typical rodent faecal pellet (dry weight approximately 10 mg) was, after hydration, able to conduct electricity and burn rapidly during REIMS acquisition (Fig. 1b; a video file of the burn process is given in Additional file 1: Video S1). Each pellet burn (Fig. 1c) generated a single mass spectrum aggregated over the total burn time (Fig. 1d). The burn events for multiple faecal pellets derived from each individual (typically three or four pellets) were averaged prior to further analysis. The negative ion spectra are rich in singly charged ions, and the region from 600 to 900 m/z is likely to be dominated by phosphatidyl glycerols, phosphatidyl ethanolamines and phosphatidic acids. Other ions at lower m/z values (150–400 m/z) were likely to be derived from fatty acids, rhamnolipids, ceramides and short-chain mycolic acids. These classes of molecules have been identified previously by REIMS/tandem mass spectrometry [19, 20], although there is little prior information about lipid profiles of rodent faeces or the distribution between signals of animal or bacterial origin. The identity of the ions is not critical for these analyses, as it is the pattern of ions that are assessed. Individual pellets from a single species generate remarkably similar spectra (Fig. 1), and the spectra from different species are distinctive and consistent (Fig. 2). The raw mass spectral data are discretised by binning (typically, 0.1- or 0.05-Da bins) and then analysed by PCA, LDA or with random forests (Additional file 2: Figure S1).

### Faecal REIMS can classify species of laboratory-housed rodents

We used laboratory-housed rodents for proof of principle that REIMS could classify rodent faecal samples based on species of origin. Samples from laboratory-housed rodents reduce variation in faecal composition attributable to environmental effects, such as diet, intestinal microbiome and housing. Faecal samples were therefore collected from bank voles, field voles, wood mice, house mice and a randomly segregating cross of Wistar × Brown Norway laboratory rats, all housed under similar laboratory conditions for several months prior to sample collection. Samples were stored overnight at 4 °C before REIMS analysis. The five species generated complex mass spectra that were relatively consistent for individuals from the same species, but markedly different between the five species. The mass spectra were binned using Offline Model Builder software to yield a 7001-point discretised spectrum for each sample prior to the analysis by discriminant function analysis. The overall workflow is summarised in Fig. 2.

The REIMS negative ion spectra obtained from each faecal sample were complex, containing multiple ions across the range from 400 to 1200 m/z. It was also apparent that the averaged spectra differed between species, in the intensities of ions at specific m/z values and, visually, in the context of the overall profile (Fig. 2a). Using discriminant function analysis of the binned data, the five species were resolved into partially overlapping clusters (Fig. 2b). Using random forest classification, REIMS could resolve rodent species (housed under laboratory conditions) to an accuracy of 83% (random forest analysis, *n* = 94). The highest classification accuracy was for rats (95%), with only 1 of 20 rat samples misclassified as a wood mouse. The lowest classification accuracy occurred for house mice and wood mice (75% for both, Fig. 2c). As a final test of performance, we took the same spectra but randomly assigned them to five different ‘pseudospecies’ categories, and the data workflow was completely unable to resolve these categories (Additional file 3: Figure S2). These tests (randomisation test) have been applied throughout this work, and the data are provided in Additional file 3: Figure S2.

### Effect of sample storage or diet on REIMS classification accuracy

**Fig. 3:**
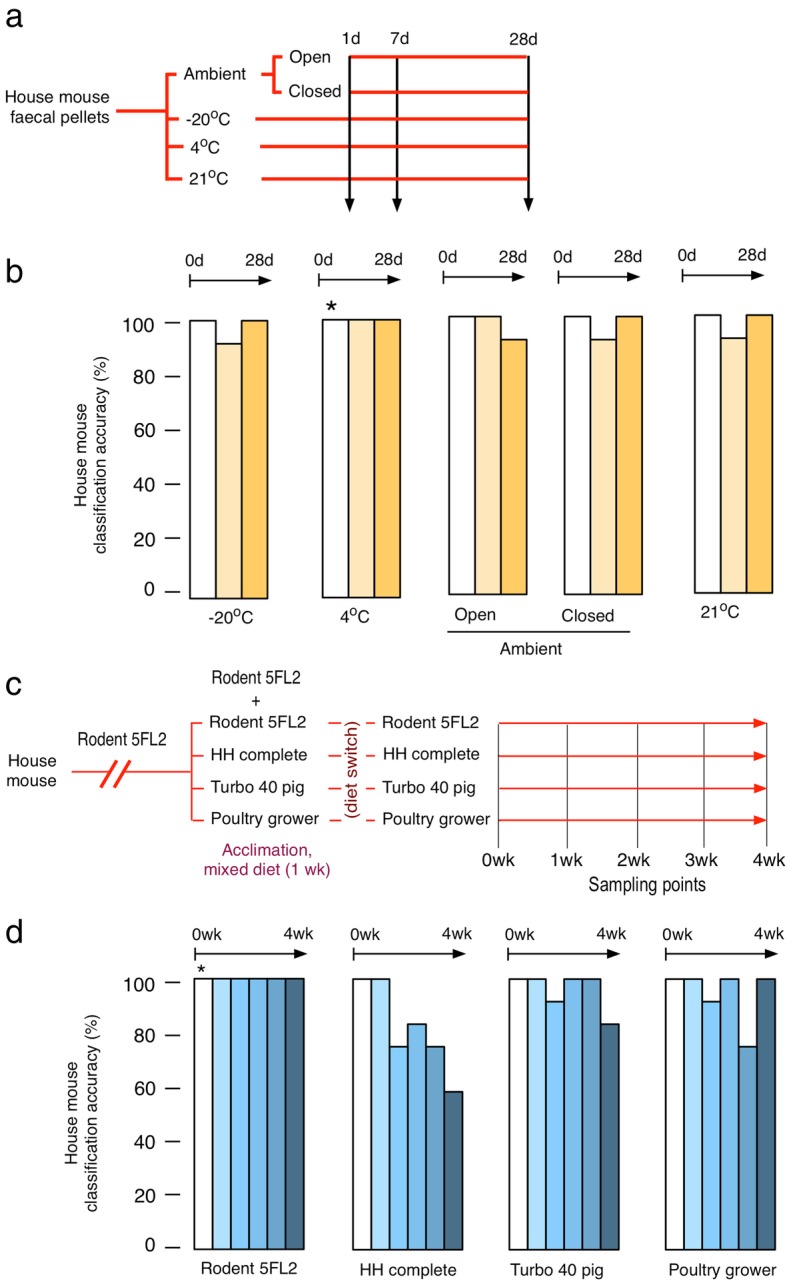
Stability of REIMS signatures to storage conditions and diet. Faecal pellets from captive-bred house mice were stored for up to 28 days under ambient temperature (average 18 °C), either in closed tubes or open to the environment. Further samples were stored for the same period in closed tubes at − 18 °C, 4 °C or 20 °C (**a**). All samples were then analysed by REIMS, and the classification accuracy under the different conditions was assessed by random forest analysis relative to baseline house mouse data taken from samples stored for 1 day at 4 °C (marked with an asterisk, **b**). To assess the effect of diet on the REIMS signature, house mice, initially fed a standard laboratory rodent 5FL2 EURodent Diet (white bars), were acclimated to a new diet over 1 week when they had access to both the old and new diets (week 0) before being transferred solely to the new diet for a further 4 weeks (**c**). Faecal pellets were collected at weekly intervals. Classification accuracy, assessed by random forest analysis, was relative to the baseline data from day 0 of the study (marked with an asterisk, **d**)

To be of greatest value, the mass signature obtained through REIMS should be stable over time and under different environmental conditions. However, there is potential for sample age and changes in ambient temperature to cause variance in the mass spectra, which could be relevant to samples collected in the field of indeterminate history and subject to a broad range of environmental conditions. We therefore explored the effect of sample history on classification accuracy. We collected faeces from captive-bred house mice and maintained them for extended periods under different conditions (Fig. 3a). The REIMS spectra from these faeces were then analysed using the random forest model established for the five species of laboratory-housed rodents (above). The stability of the signature was remarkable, and under all storage conditions, the spectra were remarkably conserved (Fig. 3a, b). No samples were misclassified for samples stored for 1 day. For samples stored for 1 week, there was a single misclassification out of 12 samples stored in closed vials at each of 3 temperatures: ambient (mean 18 °C), 21 °C and − 18 °C. For samples stored for 4 weeks, there was 1 misclassification out of 12 for samples stored in open vials at ambient temperature (mean 18 °C), conditions under which some of the faecal samples developed visible coatings of fungal hyphae. This attests to the robustness of the faecal signatures.

A second source of variation in the faecal REIMS signature could be the diet of donor animals, which would be expected to vary more in populations of wild rodents. To assess whether diet would be a major influence on the REIMS signature, we took faecal pellets from captive-bred house mice that had been maintained on standard laboratory diet and switched different groups to four different commercial diets (*n* = 12 per diet). We collected samples over several weeks as the mice were transitioned to the new diet. House mice maintained on the same diet were classified to 100% accuracy for all time points measured. Overall, for the other diets, the accuracy of classification decreased by 16% over the 5-week period, indicating that diet influences the signature, but is not a major source of variation (Fig. 3c, d). Most of the decline was due to mice transferred to a hamster diet, with three misclassifications after 1 week fully on this diet, two after 2 weeks, three after 3 weeks and five after 4 weeks (total 21.7% misclassifications). The hamster diet is an inhomogeneous mixture, and it was possible that individuals exerted some selectivity in the components of the diet that were ingested. No individual was misclassified more than twice. Only 7 out of 120 faecal samples (5.8%) were misclassified from mice on homogeneous pig or poultry diets. Thus, and perhaps surprisingly, diet was not a major contributor to the REIMS signature, suggesting that the ions are derived from host cells sloughed off in the faecal pellet or from bacteria within the relatively stable faecal microbiome.

### Faecal REIMS can classify wild rodents with a high degree of confidence

**Fig. 4:**
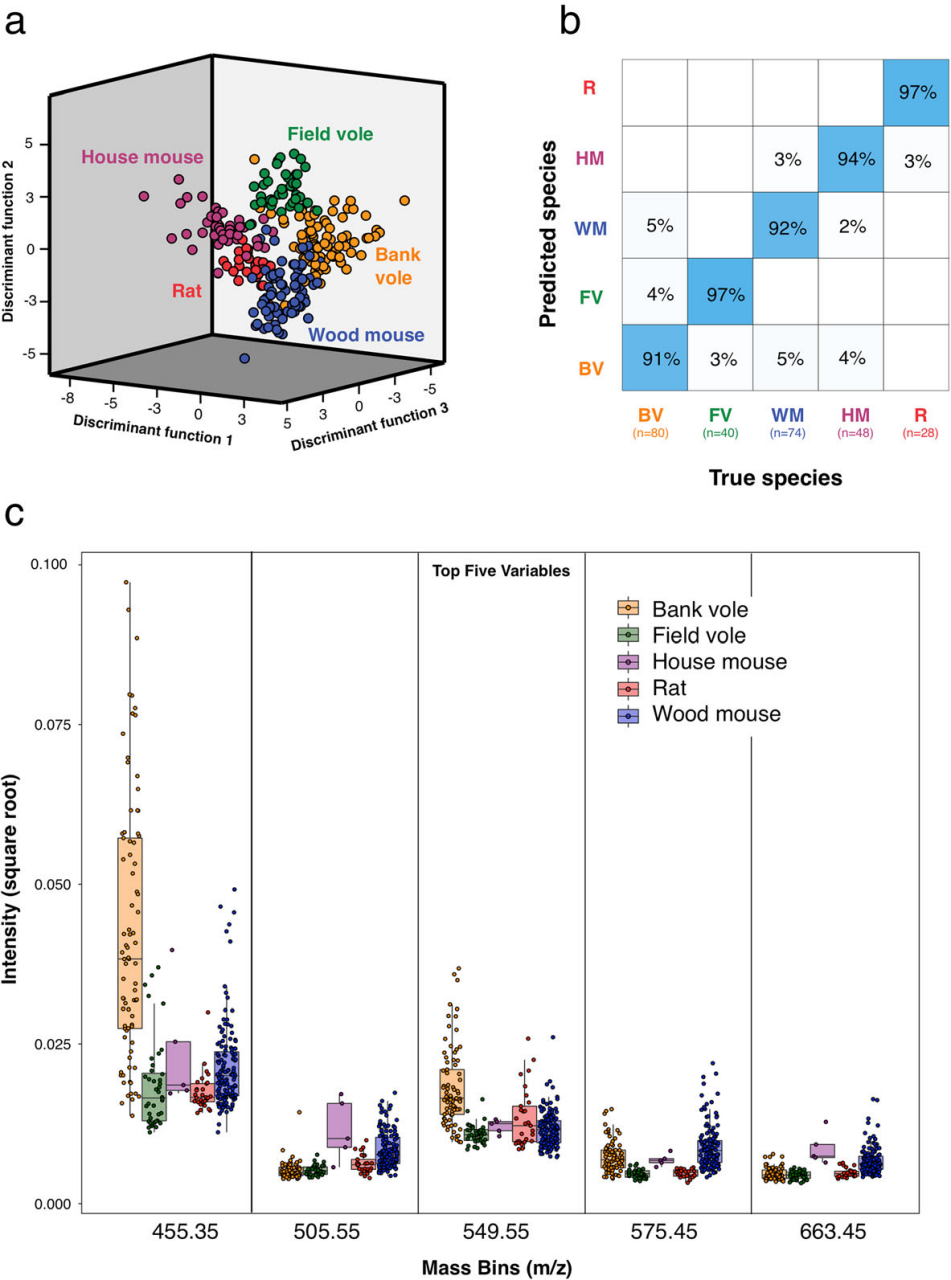
REIMS species classification accuracy for wild rodents. REIMS data were collected for faecal pellets from wild-caught rodents (28–80 individuals per species). House mice were captive-bred but maintained on one of four different diets. **a** Discriminant function analysis based on the top 12 principal components for classification of bank voles (BV, orange points), field voles (FV, green points), house mice (HM, purple points), wood mice (WM, blue points) and Norway rats (R, red points). **b** Random forest confusion matrix for the classification of species. **c** The intensity values of the five most discriminant ion bins in the mass spectra

Having confirmed that REIMS could classify rodent faeces samples from laboratory-housed animals, with a very limited effect of diet or sample storage on classification ability, we next analysed field-collected samples from bank voles, field voles, wood mice and Norway rats gained from several sites. As we were unable to capture free-living house mice for inclusion in this analysis, we used samples taken at the end of the captive-bred house mouse diet study to reflect house mice on a range of diets typical of commensal habitats. REIMS could classify and identify the correct rodent species to a high accuracy of 91 to 97% (Fig. 4a, b). For these analyses, we plotted the intensity of the five m/z bins that yield the strongest discrimination in the random forest analysis (Fig. 4c). However, in some instances, we noticed a strong cross-correlation between m/z bins separated by 1 Da, reflecting the existence of the monoisotopic ion and the first ^13^C isotopomer (example in Additional file 4: Figure S3); in these instances, we did not remove one of the ions from the analysis but have not displayed the intensity values for both ions, displaying only the monoisotopic. Other ion pairs, separated by 1-Da bins, were also correlated but did not show the expected relationship of intensity, suggesting that they are derived from the same molecular class, but are not ^12^C/^13^C pairs. These plots illustrate the broad range of ion intensity values obtained from individual faecal pellets and the considerable overlap between the spectra from each of the five species. Despite this, discrimination was highly successful—whereas a randomisation test showed no classification ability (Additional file 3: Figure S2).

### REIMS can classify species drawn from populations not included in the original data set

For REIMS to be of value in the identification of species of rodents in new study sites, it should be possible to use pre-existing data as a learning set that extrapolates to new populations. To test this using our data set, we selected 1 of our wild rodent trapping sites (Wood Park Farm) that had yielded 25 wood mouse and 11 bank vole samples. After the removal of these samples from our wild rodent data set, we ran a random forest training model on the remaining data. This training model gave a classification accuracy of 94% (data not shown). The excluded samples were then passed through the random forest model using the predict function to classify the species of origin. The model classified all 25 wood mouse samples correctly and 8 out of 11 bank vole samples correctly.

### REIMS can discriminate mouse sex and maturity

Having established that REIMS is effective at discriminating rodent species, and that this discrimination is more powerful in wild-caught than laboratory-housed rodents, we asked whether the analytical methodology could discriminate different classes of animals within the same species. These studies were conducted with three strains of laboratory mice (outbred ICR (CD-1) and inbred C57BL/6 and BALB/c). Faecal samples were collected from sexually mature (> 36-day female, > 52-day male) and immature mice of both sexes from all three strains.

**Fig. 5:**
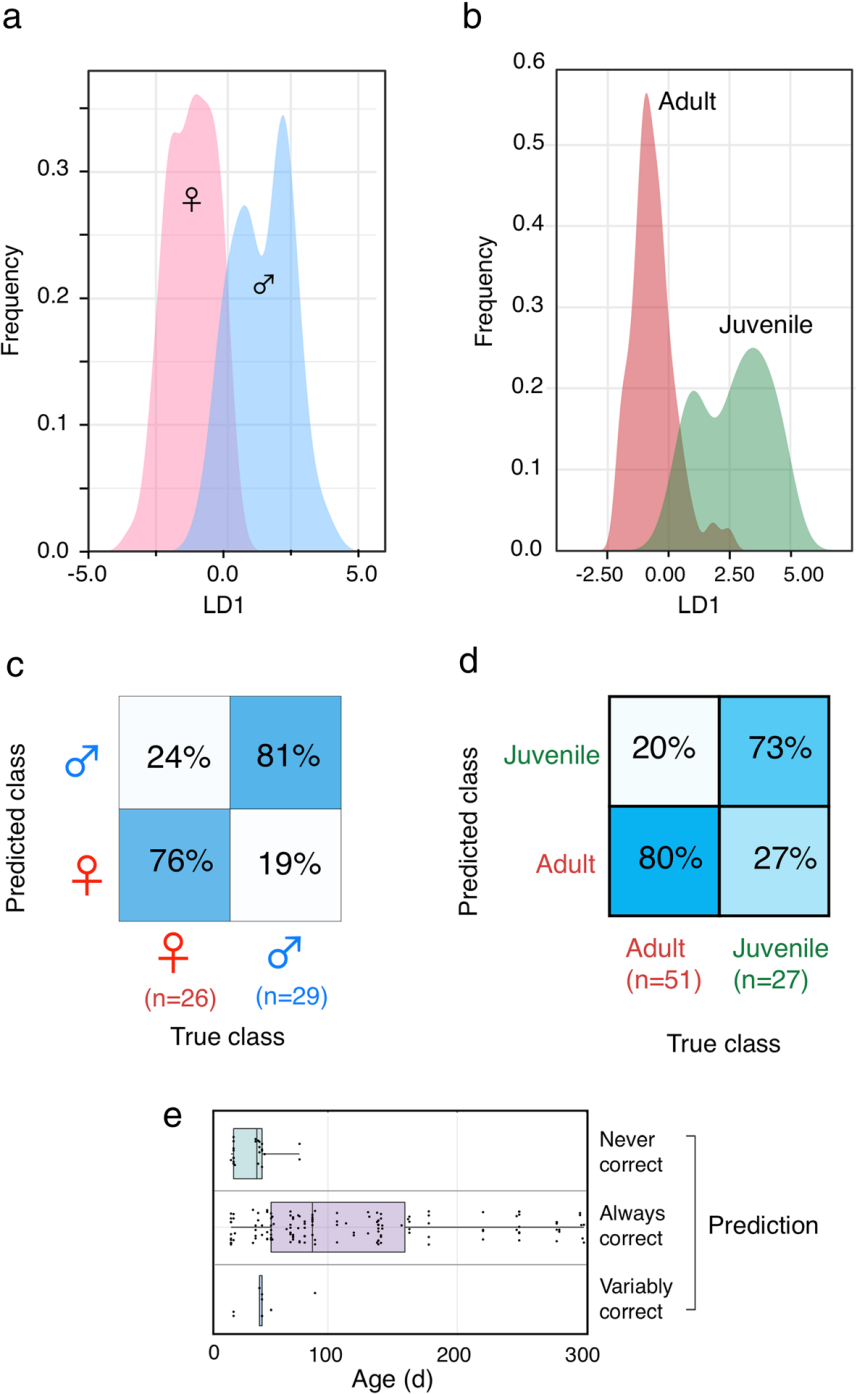
REIMS species classification accuracy for sex and maturity. REIMS spectra were acquired for faecal samples from sexually mature and immature mice of both sexes, and the entire data set was analysed in terms of the ability to discriminate sex or maturity. The distribution of the linear discriminant function for sex (**a**) and age (**b**) indicates the overlap between the categories, and the performance matrices (**c, d**) summarise the performance statistic using random forest classification. For the classification of age, the faecal samples were categorised in terms of the quality of the prediction, based on 10 random forest constructions (**e**)

**Fig. 6:**
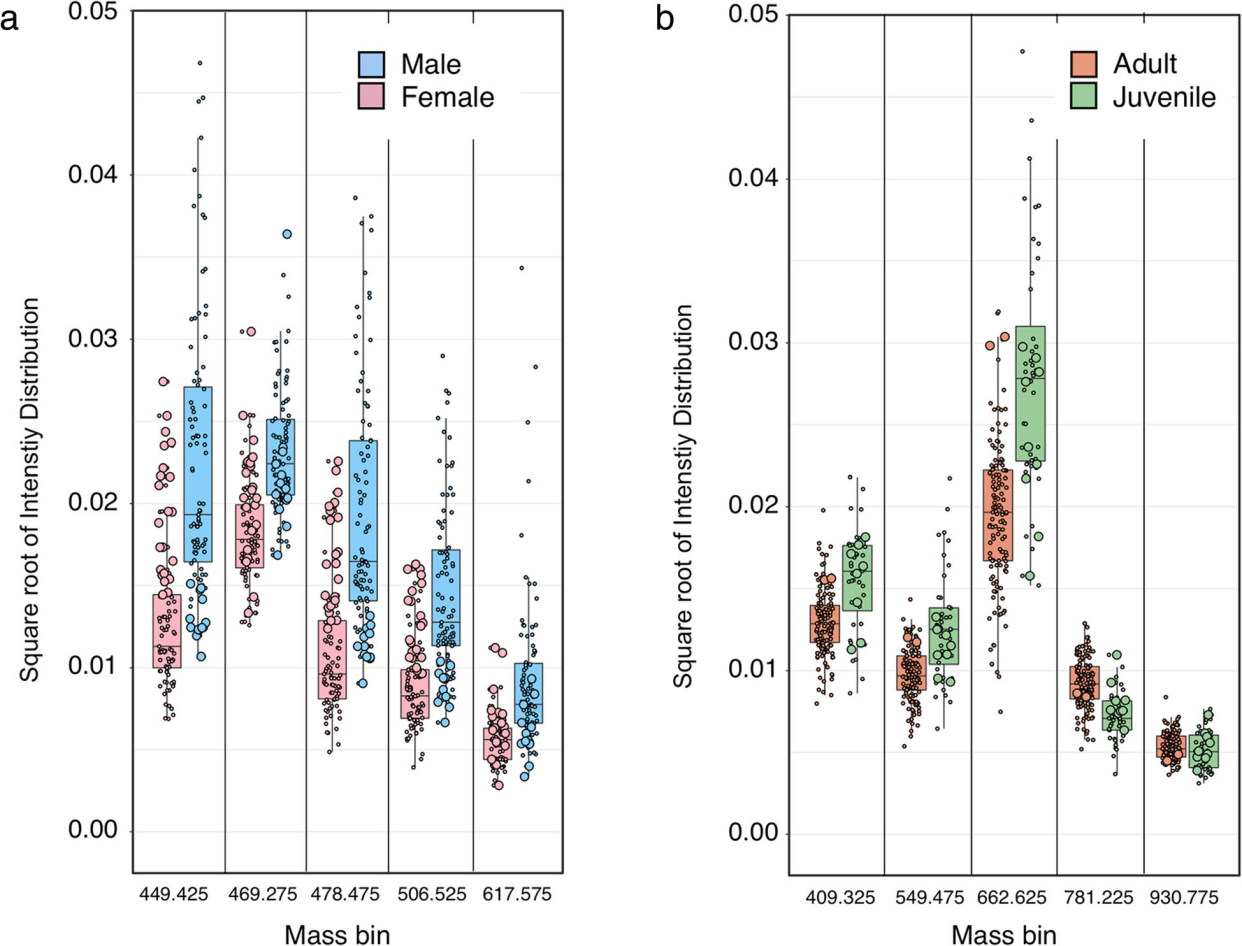
Mass to charge bins for discrimination of sex and age. Negative ion REIMS mass spectra were acquired for faecal samples from adult and juvenile mice of both sexes, and the entire data set was analysed in terms of the ability to discriminate sex or age. The intensities of the five most informative m/z bins, derived from ‘random forest explainer’, are plotted, together with summary statistical plots (median, interquartile ranges, 5–95% whiskers, individual values) for sex (**a**) and age (**b**) discrimination. In each plot, large symbols represent the intensity values for individuals that were misclassified, and small symbols represent correctly classified samples

There was segregation of faecal pellets on the basis of sex (all ages) or maturity (both sexes) of the owner (Fig. 5, Additional file 3, for randomisation tests). As perhaps would be expected, the classification of sex (a categorical variable) was stronger than for maturity (based on age, a continuous variable using a single age cut-off. Analysis of misclassification based on age revealed that it was predominantly attributable to the incorrect assignment of samples from young individuals. The role of different m/z bins in discrimination is clear from a plot of the five most informative signals, nominated by the RandomForestExplainer package [21] for each discrimination (Fig. 6). There is a considerable overlap between the groups for each bin, but the bias in each is evident, and the combination of data from multiple bins provides useable discrimination. There is unlikely to be a single bin or molecular species that drives discrimination. Analysis of the intensities obtained from individual samples that were misclassified revealed that these tended to be clustered above or below the median for the category.

### REIMS can discriminate different mouse strains

**Fig. 7:**
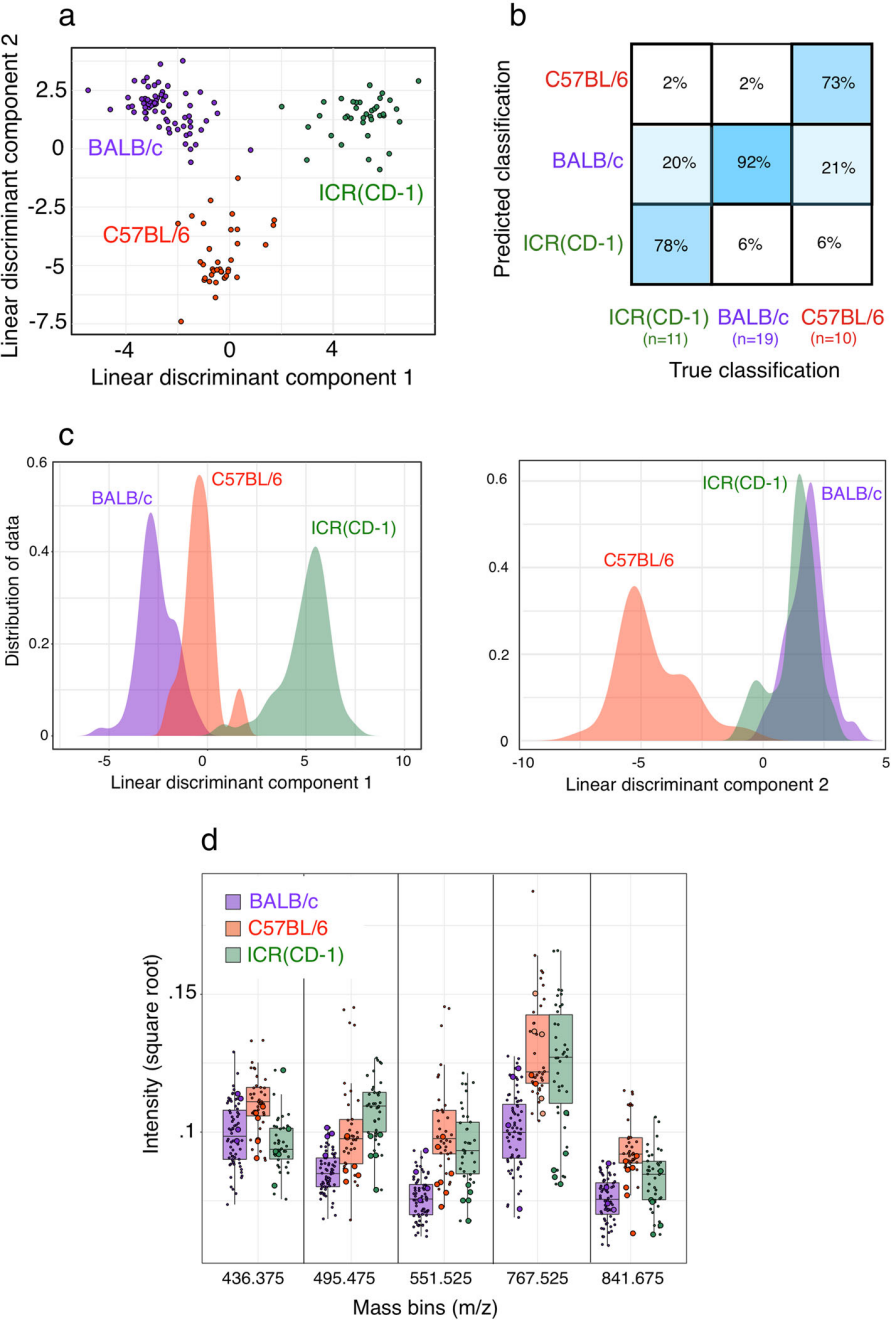
REIMS analysis of faecal samples from different mouse strains. Negative ion REIMS mass spectra were acquired for faecal samples from three mouse strains (both sexes). LDA plot of first and second components. (**a**). The performance from a random forest classification is summarised in **b**. The distribution of the first and second linear discriminant components based on the 60 most informative m/z bins derived from principal component analysis **c, d**. Intensities of the five most discriminatory mass bins that drive strain segregation; large symbols are from individual samples that were misclassified

Most laboratory mouse strains originate from common ancestors but have been isolated for many hundreds of generations, leading to multiple genotypic and phenotypic changes [22]. We therefore explored the ability of REIMS to discriminate between the faeces of three strains from different major lineages of laboratory mice [22]. These were two inbred strains (BALB/c, derived from the Castle lineage, and C57BL, derived from the C57 lineage) and ICR random-bred mice derived from the Swiss lineage. All were fed on the same diet and kept under the same husbandry conditions. Complex spectra were obtained from all three strains, with clear differences being evident. This confirms that REIMS is capable of a high level of discrimination within species as well as between species (Fig. 7).

## Discussion

We have demonstrated that REIMS can generate informative mass spectra from rodent faeces, and these can be used to classify species of origin with high performance (typically > 90%). The REIMS signature does not comprise a series of identified molecular ions, identified by their mass spectra. Rather, the pattern of ions, many of which reflect chemical modification in the REIMS acquisition, are uninterpreted in molecular terms, and it is the overall signature that is used for discrimination. The species discrimination when applied to faecal samples collected from wild-caught animals by live trapping was very high (between 91 and 97%) and superior to that obtained with animals maintained in the laboratory. This increased discrimination of wild samples could be a consequence of a broader range of dietary variation. Wild rodents have a heterogenous diet [2], and it is perhaps unsurprising that species maintained on a common diet were slightly less strongly discriminated. However, even with a homogenous diet, the ability to discriminate all species was remarkable. The loss of classification accuracy observed when house mice were maintained on a hamster diet could be due to this being the most varied of the four diets we used, leading to a greater variation in dietary intake by individual mice due to preferential selection of specific diet components. Discrimination based on faecal components may be further enhanced by appropriate choice of an optimal learning algorithm [23]; these data are already sufficiently encouraging to explore further application in field ecology.

The overall resilience of the REIMS signature suggests that a major component of this signature is attributable to the animal, possibly due to species-specific compounds excreted in the faeces. Such compounds may, for example, originate from anal gland secretions that have been previously identified in rodent faeces [24–26]. It may also be possible that shifts in the gut microbiome elicited by dietary differences could manifest in the faecal molecular profile. Thus, the REIMS signature may be composed of a combination of species-specific elements, and additional elements influenced by other factors, such as diet. Overall, the high classification accuracy of wild rodent faecal samples, compared to those from laboratory-housed rodents, bodes well for future field studies. Further, it was reassuring that the REIMS profile was robust to sample storage. Field-collected samples could be retained and analysed at the conclusion of a field study, for example. This contrasts with the known instability of specific faecal metabolites [27, 28]. Indeed, in the absence of a freezer, the best storage solution might be to dry the faecal samples rapidly and rehydrate them just prior to REIMS.

REIMS has considerable potential in molecular scatology. Species identification is comparable to other faecal-based identification techniques [1, 7, 29] and higher than different techniques such as photography and footprint identification [30–32]. A real benefit of REIMS is the high speed of sample processing, typically 5–10 s per burn event, with a maximum processing time of no more than 2 min per sample, much faster than DNA-based protocols which can take hours or days [10]. Although the core instrumentation necessary for REIMS is costly, it is inexpensive to build comprehensive profiles of large numbers of samples from a REIMS-based workflow, and the signatures incorporated into the learning set are robust and useable for future analyses.

The ease with which REIMS spectra are acquired, and indeed, the lack of any requirement for detailed molecular interpretation of the spectra means that this method might be applicable to many ecological applications, including conservation and biological research. Although we have combined pellets in this study, this might not feasible in field acquired samples. However, it is possible to acquire perfectly useable spectra from single pellets. Indeed, spectra can be generated from one half, or even one quarter, of a 10-mg mouse pellet (Additional file 5, Figure S4). Although samples must be brought to the instrument, the stability of the signature to storage makes this feasible. At hundreds of samples per day, it would be possible to develop a detailed profile over significant geographical or temporal scales. This could allow REIMS to be used as a central diagnostic service to which samples are sent for commercial pest control, conservation and research applications. We have already shown that REIMS can discriminate sex and maturity, and it is possible that other factors, such as stress, could also be added to the phenotypic characterisation.

## Methods

### Laboratory-housed rodent sample collection

Test subjects were 10 male and 10 female bank voles (*Myodes glareolus*), 8 male and 10 female field voles (*Microtus agrestis*), 6 male and 10 female wood mice (*Apodemus sylvaticus*), 10 male and 10 female wild-stock house mice (*Mus musculus domesticus*) and 10 male and 10 female laboratory rats (*Rattus norvegicus*). Bank voles and field voles were a mixture of animals wild-caught in Northwest England 9 to 15 months prior to the start of the study and first-generation offspring of individuals wild-caught in Northwest England aged 5 to 18 months. Wood mice were all wild-caught in Northwest England 22 to 27 months prior to the start of the study. House mice were 9 to 17 months old, captive-bred for 5–10 generations from populations captured in Northwest England. Inbred or random-bred laboratory strains were Hsd:ICR (CD-1®, ‘ICR’), C57BL/6JOlaHsd (C57BL/6) and BALB/cOlaHsd (BALB/c) were in-house bred. Rats were 8 to 9 months old from a random-bred cross between Wistar (HsdHan®:WIST, InVivo, Bicester, UK) and Brown Norway (BN/SsNOlaHsd, InVivo, UK) laboratory strains, originally obtained from Envigo UK and subsequently crossbred in-house for three generations. Bank voles, field voles and male house mice were housed singly in 48 × 15 × 13 cm cages (M3, North Kent Plastics, UK). Female house mice were housed in groups of 2 to 4 full siblings in 45 × 28 × 13 cm cages (MB1, North Kent Plastics, UK). Wood mice were housed singly in 38 × 25 × 18 cm cages (RM2, North Kent Plastics, UK). Rats were housed in same-sex pairs in 56 × 38 × 22 cm cages (RC2R, North Kent Plastics, UK).

All animals were fed 5FL2 EURodent Diet (IPS Product Supplies Limited, London, UK) ad libitum and had access to water ad libitum. Wood mice, bank voles and field voles were supplemented with Harry Hamster complete muesli (Supreme Petfoods Ltd., Ipswich, UK) and hay. Field voles were also given fresh-cut grass. All cages had Corn Cob Absorb 10/14 substrate (IPS Product Supplies Limited, London, UK) lining the base. Cardboard tubes and paper wool nest material were provided to all animals for enrichment, with 15 × 8 cm plastic tubes also provided for rats. Animal numbers are summarised in Additional file 6: Table S1.

### Collection of faecal pellets

Laboratory-housed rodents were placed individually into a clean laboratory cage for 1 to 2 h, and multiple faecal pellets were collected from each individual. The order of sample collection from each animal was randomised.

### Wild rodent sample collection

Longworth traps, Mk1 or Mk2 TubeTraps (BioEcoSS Ltd., Shropshire, UK) and Ugglan traps were set in five separate locations: Kielder Forest (Northumberland, UK); Ness Botanic Gardens (Wirral, UK); Wood Park Farm (Wirral, UK); University of Liverpool, Leahurst Campus (Wirral, UK); and a private garden in Mouldsworth (Cheshire, UK); locations are recorded in Additional file 6: Table S1. Traps, cleaned before every use, were baited with parakeet seed mix (Rob Harvey, Tongham, UK) and a piece of apple. Hay was provided in the traps as bedding material. Traps were checked twice daily. Species and sex of all trapped animals were recorded, and multiple faecal pellets were taken from each trap. Sex was determined using anogenital distance. No faecal samples were taken when more than one animal was captured in the same trap, and animals were fur clipped to avoid repeated sampling. Faecal samples from wild Norway rats (*Rattus norvegicus*) were obtained as loose droppings from building floors at Wood Park Farm (Wirral, UK), Ness Heath Farm (Wirral, UK) and Shotton Industrial Estate (Wirral, UK). The distance between the sample locations and local population density ensured that samples were very likely to be from different individuals. For the discrimination study for wild rodents, samples were collected from 80 bank voles, 40 field voles, 74 wood mice and 29 rats (Additional file 6: Table S1) and stored for up to 15 days at − 20 °C before the analysis.

### Evaluation of storage conditions

Faecal donors were 12 male captive-bred house mice (bred for 5–10 generations from populations captured in Northwest England) aged 11 to 13 months. Multiple faecal samples were collected from each donor and stored in closed Eppendorf tubes at 4 temperatures: − 18 °C, − 4 °C, 21 °C and ambient (mean 18.25 °C, maximum 24 °C, minimum 17 °C). Ambient temperature samples were stored in open or closed Eppendorf tubes. Samples were stored for 1 day, 1 week or 4 weeks. Samples were randomly allocated to each temperature and time condition. For each donor, a sample was stored for each temperature and time condition, giving 15 samples per donor.

### Diet study sample collection

Test subjects were 48 singly housed wild-stock male house mice (9 to 18 months old, bred for 5–10 generations from populations captured in Northwest England). All subjects were fed 5FL2 EURodent Diet ad libitum prior to the start of the study. At the start of the study, mice were assigned to four treatment groups (*n* = 12) and fed different diets. During an acclimation week, subjects were fed a mixture of 5FL2 EURodent Diet and their new diet. From the second week, house mice were fed only their new diet for a further 4 weeks. Diets were Poultry Grower (SDS, Braintree, UK), Harry Hamster complete muesli (Supreme Petfoods Ltd., Ipswich, UK), Turbo 40 pig feed (Massey Bros Feeds Ltd., Crewe, UK) and 5FL2 EURodent Diet (IPS Product Supplies Limited, London, UK). Faecal samples were collected from each mouse on the first day of the study and at weekly intervals over the study.

### Sex, maturity and strain study

Test subjects came from two inbred laboratory mouse strains, C57BL/6JOlaHsd (C57BL/6) and BALB/cOlaHsd (BALB/c), and one random-bred laboratory strain, Hsd:ICR (CD-1®, ‘ICR (CD-1)’). The strains were originally obtained from Envigo UK and subsequently bred in-house. They were maintained in MB1 cages, and faecal samples were collected by temporary transfer to M3 cages. All animals were fed on 5FL2 EURodent Diet and had access to water ad libitum*.* All cages contained Corn Cob Absorb 10/14 substrate, 15 × 5 cm plastic tubes and paper wool nest material. Samples were collected from 176 individuals (details in Additional file 6, Table S1; BALB/c and BALB.K were combined for this study). Samples were stored at 4 °C for up to 7 days or at − 18 °C for up to 30 days prior to analysis.

### REIMS processing of faecal samples

All sampling was conducted in a Ductless Fume box (Air Science, Liverpool, UK). REIMS requires that samples contain sufficient water to conduct an electric current to heat the sample and generate fumes. As faecal pellets collected in the field may have dried to a variable degree, we optimised a rehydration protocol. Faecal pellets were placed onto 25-mm glass microfiber filter paper disc (GE Healthcare/Whatman), moistened with MilliQ water. The pellets were then individually hydrated with 200 μL of MilliQ water for 1 to 2 min. An aerosol was generated using a monopolar electrosurgical pencil in either cut mode at 35 W (species discrimination) or coagulate mode at 40 W (mouse age, sex, strain) powered by a VIO 50 C electrosurgical generator. Sampling was of three to five pellets from the same individual and/or condition for data acquisition for 2–5 s per pellet. Sample processing was conducted blind to the treatment condition of the sample, and the order of sample processing was randomised.

Aerosol particles were aspirated using a Venturi gas jet pump powered by nitrogen on the REIMS source via a 3-m evacuation tubing incorporated into the electrosurgical pencil. The Venturi pump introduces the aerosol orthogonally to the inlet capillary of the mass spectrometer, which is then drawn into the source by the vacuum of the instrument. This geometry, combined with a specially designed whistle within the Venturi housing, ensures that the larger particles are not drawn into the capillary where they could cause a blockage. A solution of leu-enkephalin (1.72 pmol/μL dissolved in propan-2-ol) (Fisher Scientific) was infused at 100 μL/min and nebulised at a position opposite the inlet capillary within the whistle assembly. This peptide was used as a lock mass (544.26 m/z) to maintain an accurate mass measurement during all analyses. Laboratory animal and storage studies were conducted using the beta version of the impactor (ceramic cylinder) whereas the wild animal and diet study samples were analysed using the commercial version (Kanthal metal coil). Mass spectra were recorded on a Synapt G2-Si (Waters, Wilmslow, UK) in full-scan resolution, negative ion mode at a scan rate of 1 scan per second from 50–1200 m/z. The sample cone was set to 60 V, and the heater bias was set to 60 V.

### Data analysis

An overview of the data analysis workflow is presented in Additional file 2: Figure S1. Individual burn spectra for each faecal pellet were aggregated to generate a single raw data file for each sample. Mass spectra were imported into Waters Offline Model Builder software (OMB-1.1.28, Waters Research Centre, Hungary) or LiveID (Waters). Within Offline Model Builder spectral data above, the intensity threshold of 3 × 10^5^ counts were summed for each data point, accumulating data from multiple faecal pellets from the same animal. Within LiveID, intensity threshold was set automatically, and the exported, binned data were further processed in R. Mass spectra were then lockmass corrected to either a propan-2-ol background peak at 325.19 m/z or leu-enkephalin at 554.26 m/z. For analysis, a mass range of 400 to 1100 m/z was used. The resulting spectra were normalised, scaled and binned by either LiveID or Offline Model Builder (Waters) at 0.05 or 0.1 m/z bin width. Binned data (approx. 14,000 or 7000 data points) were exported as .csv data files for further analysis. For some experiments, data were analysed by principal component analysis (PCA) followed by discriminant function analysis (DFA) using either SSPS version 24 (IBM, Portsmouth, UK) or R. Random forest classification was achieved with package ‘randomForest’ [33] using R version 3.4.2. [34]. For random forest analysis, two analyses were completed—in the first, all samples were included in the classification, and in the second, we retained 70% as a training set and used the trees generated therefore to assess the remaining 30%. A confusion matrix was generated to determine the accuracy of classification for each species or another category. Specific ions that made the greatest contribution to classification were identified using the randomForestExplainer package [21] in R. Data were visualised with SPSS or with ‘ggplot2’ in the R environment [35]. In some instances, the top informative ions included both the monoisotopic ion and the first ^13^C isotopomer that were identified and confirmed by plotting a cross-correlation matrix for the intensities of these ions—such isotopically linked pairs exhibited a very high degree of correlation, as would be expected (Additional file 4: Figure S3).

## Additional files

Additional file 1: **Video S1.** The consumption of a faecal pellet by diathermy in the REIMS process. (GIF 20119 kb)

Additional file 2: **Figure S1.** Overall data acquisition and processing workflow. (PDF 930 kb)

Additional file 3: **Figure S2.** Randomisation tests based on random assignment to classes. (PDF 326 kb)

Additional file 4: **Figure S3.** Ion intensity cross-correlation analysis. (PDF 65 kb)

Additional file 5: **Figure S4.** Demonstration of REIMS spectra derived from one half, or one quarter, of mouse faecal pellets. (PDF 2071 kb)

Additional file 6: **Table S1.** Numbers of donors of faecal samples used in this study. (PDF 46 kb)

## Data Availability

All REIMS raw data files are available in the MetaboLights database [36]. Accession number: MTBLS1095.
