# Supplementary figures and images for: Rapid identification of species, sex and maturity by mass spectrometric analysis of animal faeces
Source: BMC Biol. 2019 Aug 14;17:66. doi: 10.1186/s12915-019-0686-9 (PMC6693146; doi:10.1186/s12915-019-0686-9)

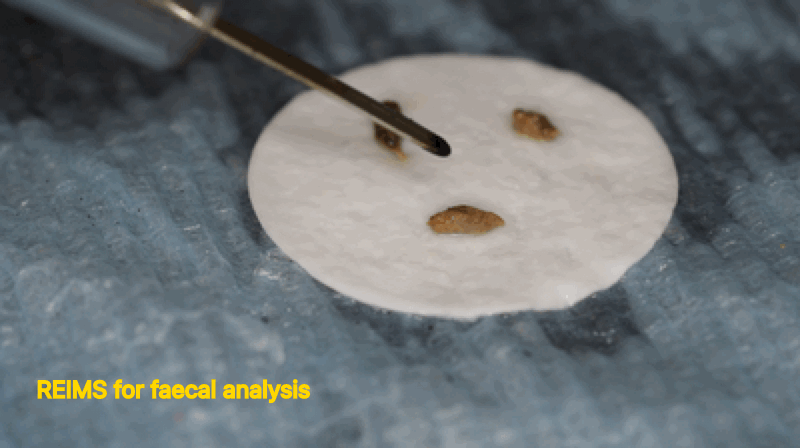

Supplement: Supplementary file 1 — Video S1. The consumption of a faecal pellet by diathermy in the REIMS process. (GIF 20119 kb) [file 12915_2019_686_MOESM1_ESM.gif]
