# Supplementary material for: Rapid identification of species, sex and maturity by mass spectrometric analysis of animal faeces
Source: BMC Biol. 2019 Aug 14;17:66. doi: 10.1186/s12915-019-0686-9 (PMC6693146; doi:10.1186/s12915-019-0686-9)
Supplement: Supplementary file 2 — Figure S1. Overall data acquisition and processing workflow. (PDF 930 kb) [file 12915_2019_686_MOESM2_ESM.pdf]

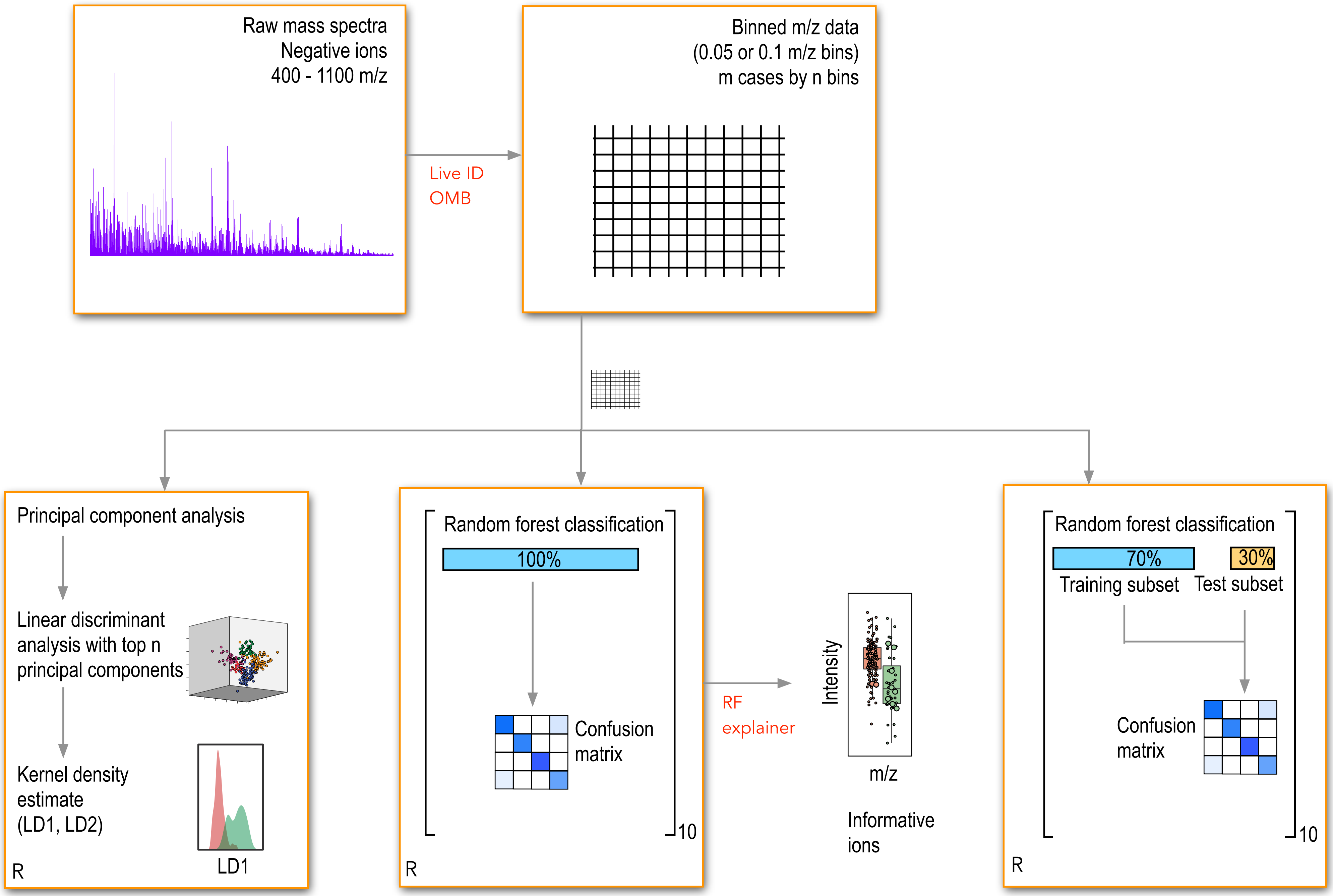

Raw mass spectra
Negative ions
400 - 1100 m/z
Live ID
OMB
Binned m/z data
(0.05 or 0.1 m/z bins)
m cases by n bins
Principal component analysis
Linear discriminant analysis with top n principal components
Kernel density estimate (LD1, LD2)
LD1
R
Random forest classification
100%
Confusion matrix
10
R
RF explainer
Intensity
m/z
Informative ions
Random forest classification
70%
30%
Training subset
Test subset
Confusion matrix
10
R

Supplementary Figure 2: Randomisation tests

Number of Individuals Used For The Randomised Classifications

Species determination. The lab and wild species data set was randomised using a random number generator to the same ratio as the five different species in the original data

Classification	Number of Lab Individuals	Number of Wild Individuals
A	20	79
B	17	41
C	21	5
D	20	28
E	17	138

Sex, age and strain. The samples for sex, age and strain were randomised using an online random number generator so each random class had the same number of individuals as the original data

Classification	Factor	Number of Individuals	% of total individuals
Sex	Random One	85	48
	Random Two	91	52
Age	Random One	132	75
	Random Two	44	25
Strain	Random One	38	26
	Random Two	38	47
	Random Three	68	26

Randomisation test: Entire Data Set

A random 50% of all samples including all sex, age and strain types assigned as class ‘Random One’ the other half as class ‘Random Two’ using an online random number generator. These randomly assigned classes were then used as input for random forest and LDA.

All Data

Predicted Classification	Random 1 n=88	Random 2 n=88
Random 2	50%	44%
Random 1	50%	56%

True Classification

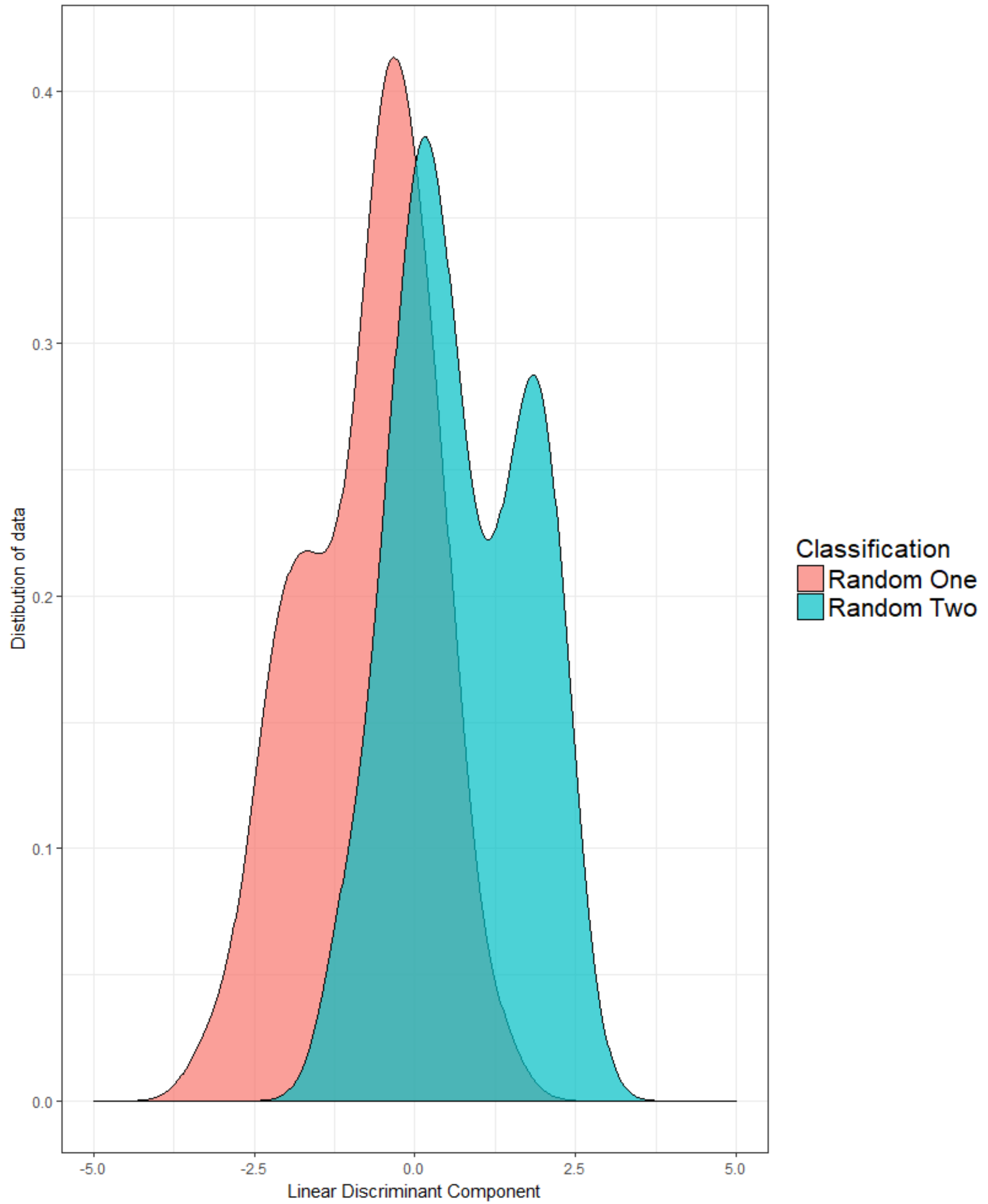

Randomisation test: sex

Randomised using the same ratio as Male and Female

All Data

Predicted Classification	True Classification: Random 1 (n=85)	True Classification: Random 2 (n=91)
Random 2	48%	51%
Random 1	52%	49%

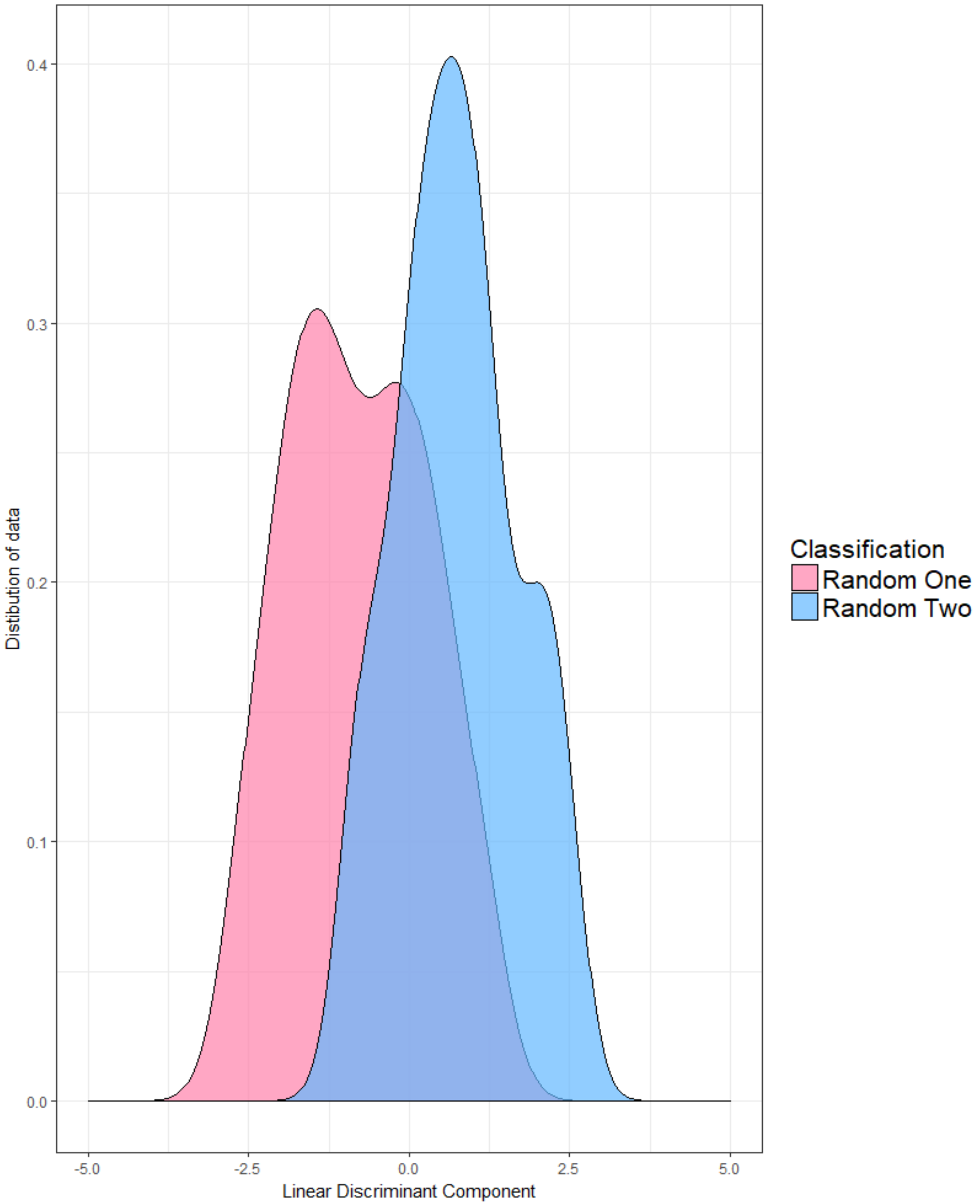

Randomisation test: age

Same ratio as Adults and Juveniles

All Data

Predicted Classification \ True Classification	Random 1 n=132	Random 2 n=44
Random 2	4%	5%
Random 1	96%	95%

Under-sampling

Predicted Classification \ True Classification	Random 1 n=44	Random 2 n=44
Random 2	61%	48%
Random 1	39%	52%

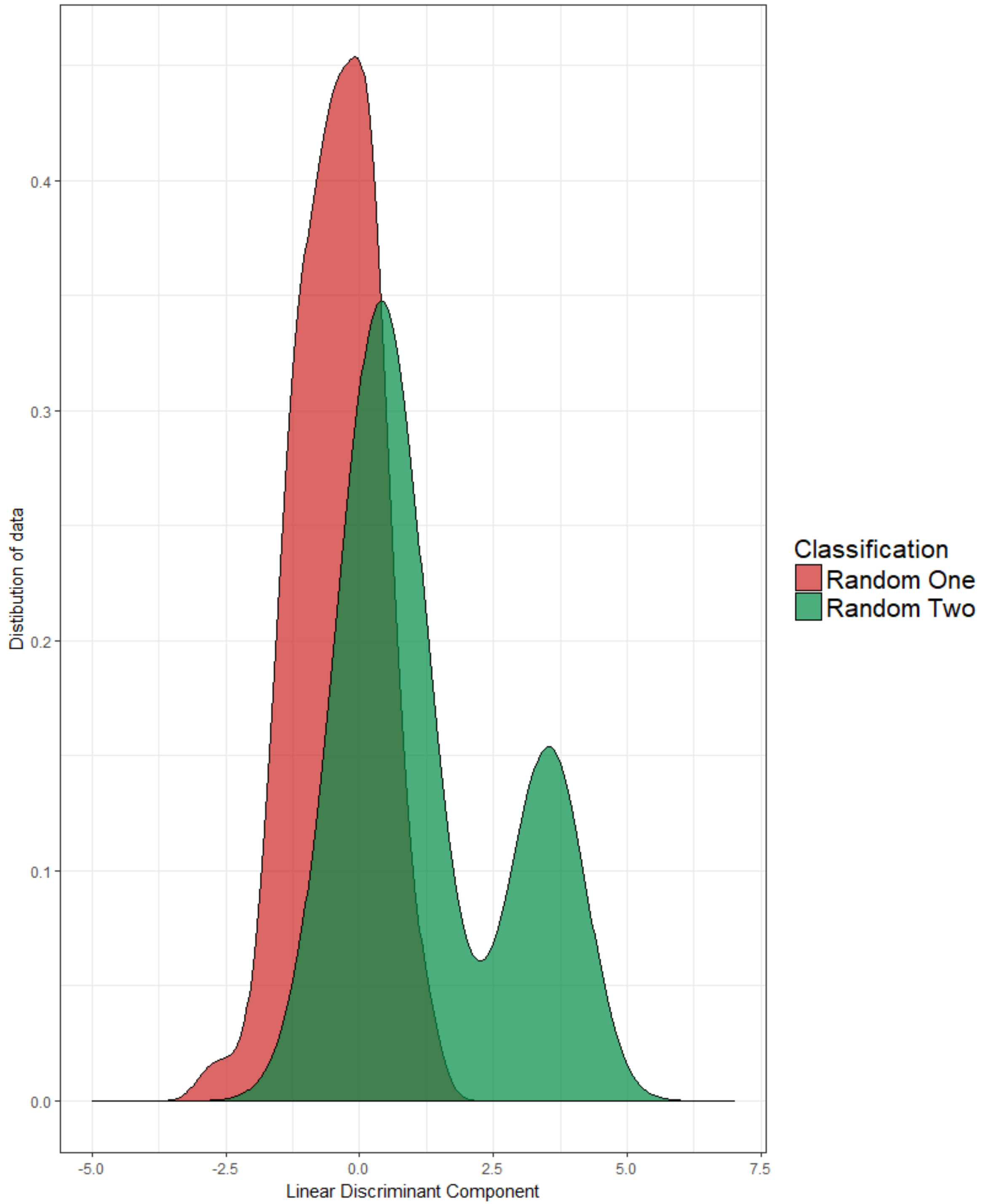

Randomisation test: sex

Ten random m/z bin were selected using a random number generator and the intensity distributions are displayed. Correct classifications: small symbols, incorrect classification: large symbols.

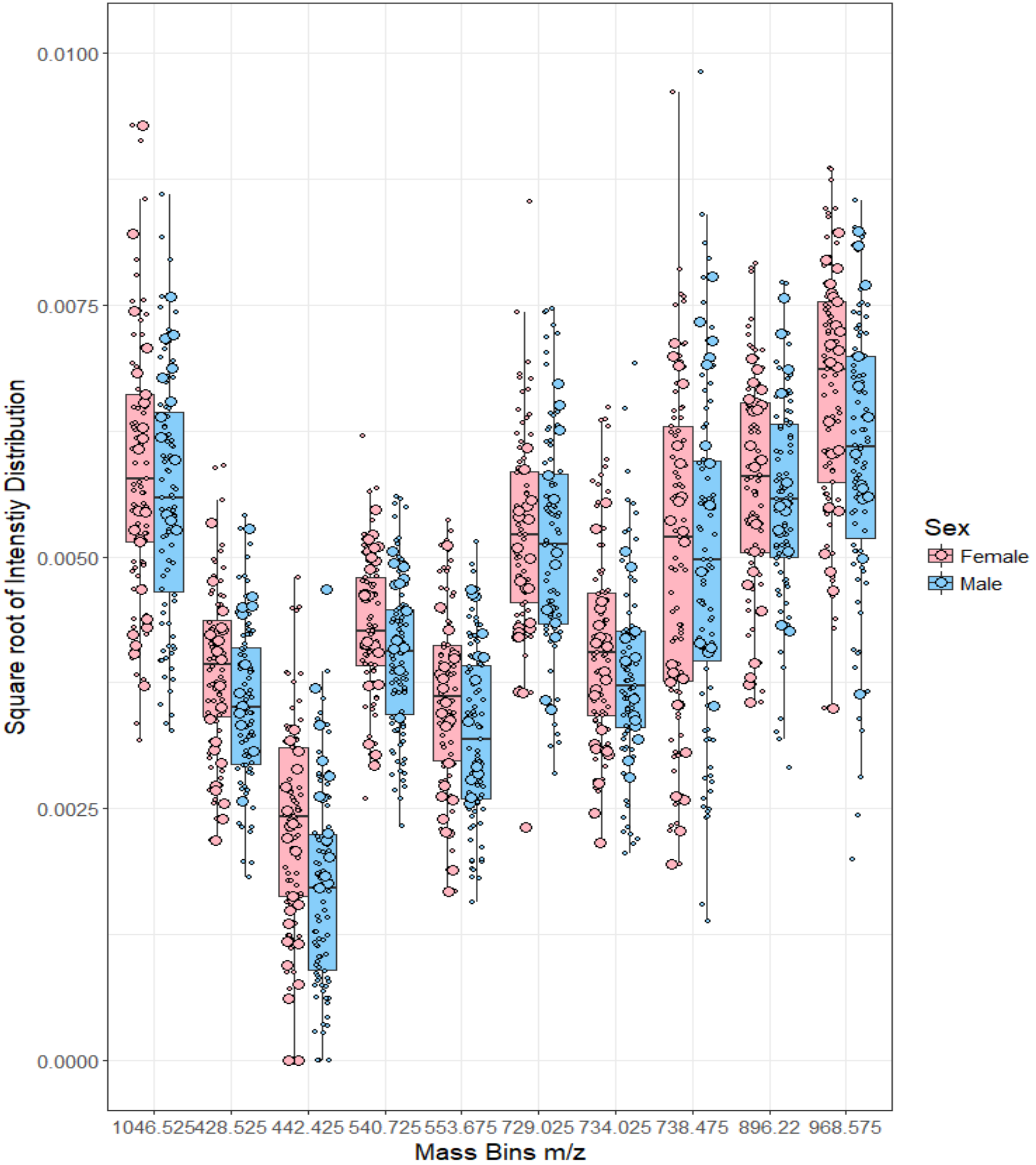

Randomisation test: age

Ten random m/z bins were selected using a random number generator and the intensity distributions are displayed. Correct classifications: small symbols, incorrect classification: large symbols.

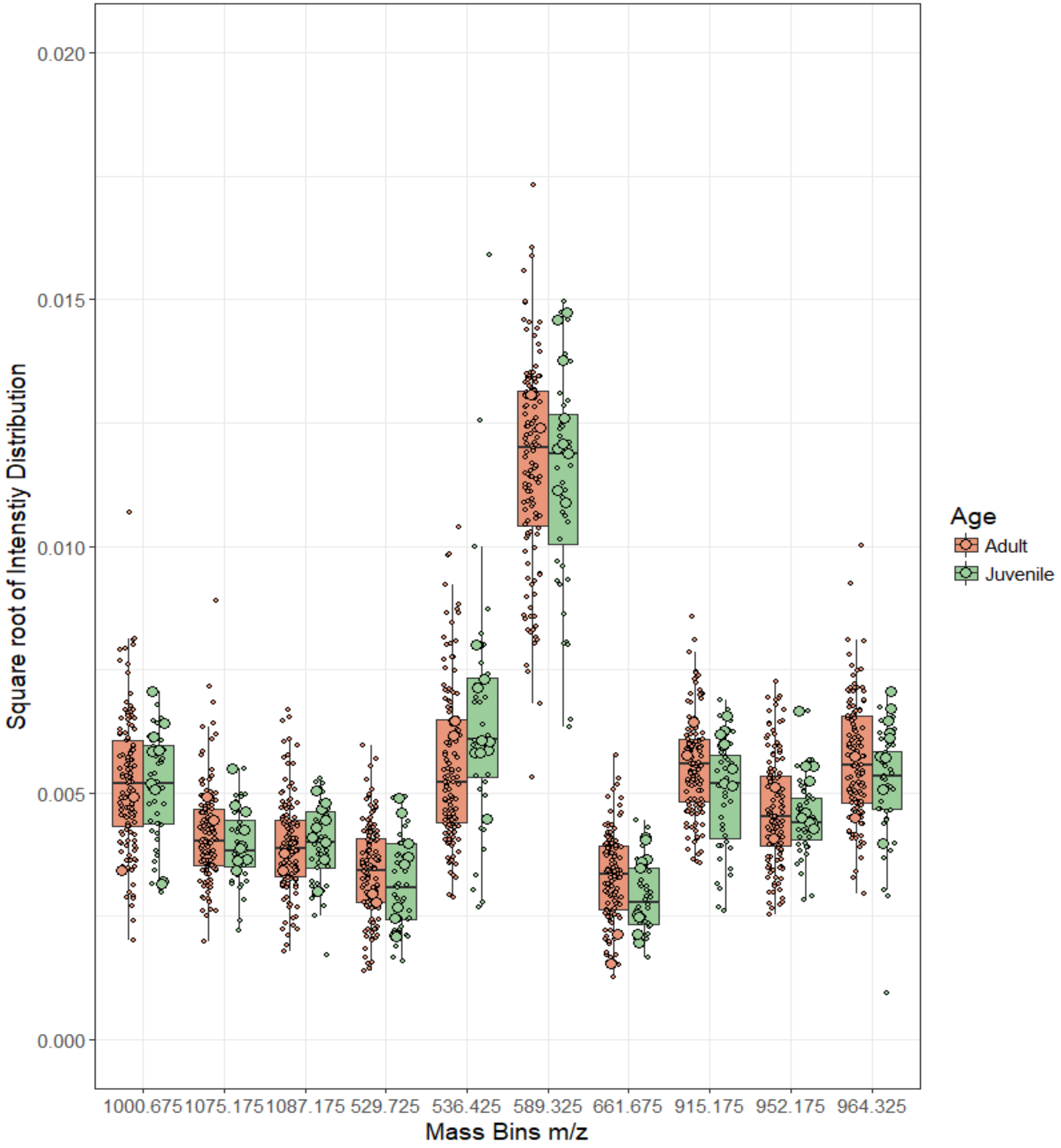

Randomisation test: strain

Samples from the three strains were randomised using the same ratio as the source samples.

All Strain Data

	Random 1 n=38	Random 2 n= 38	Random 3 n= 69
Random 3	86%	88%	81%
Random 2	6%	0%	7%
Random 1	7%	12%	12%

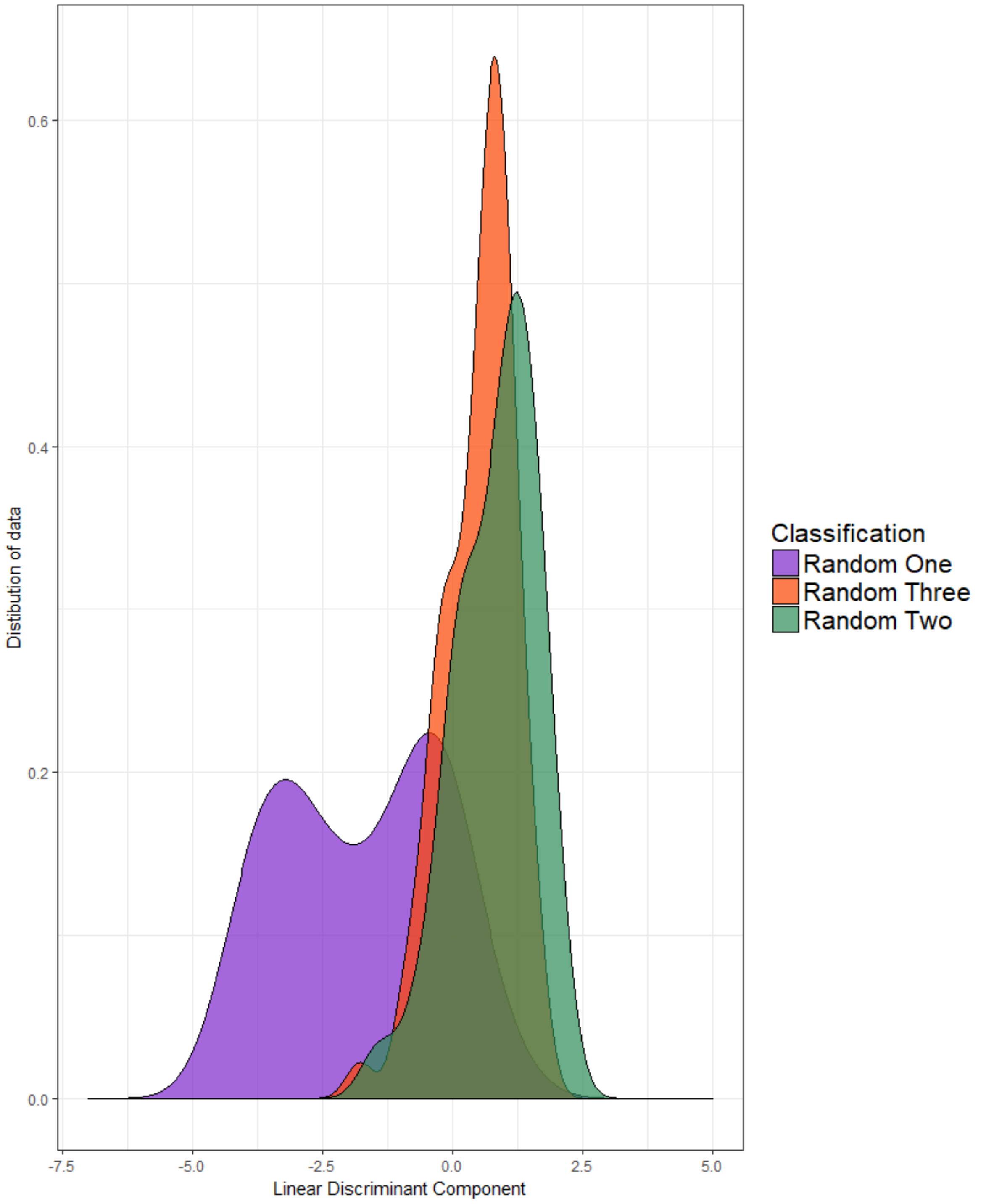

Randomisation test: strain

Ten random m/z bin were selected using a random number generator and the intensity distributions are displayed. Correct classifications: small symbols, incorrect classification: large symbols.

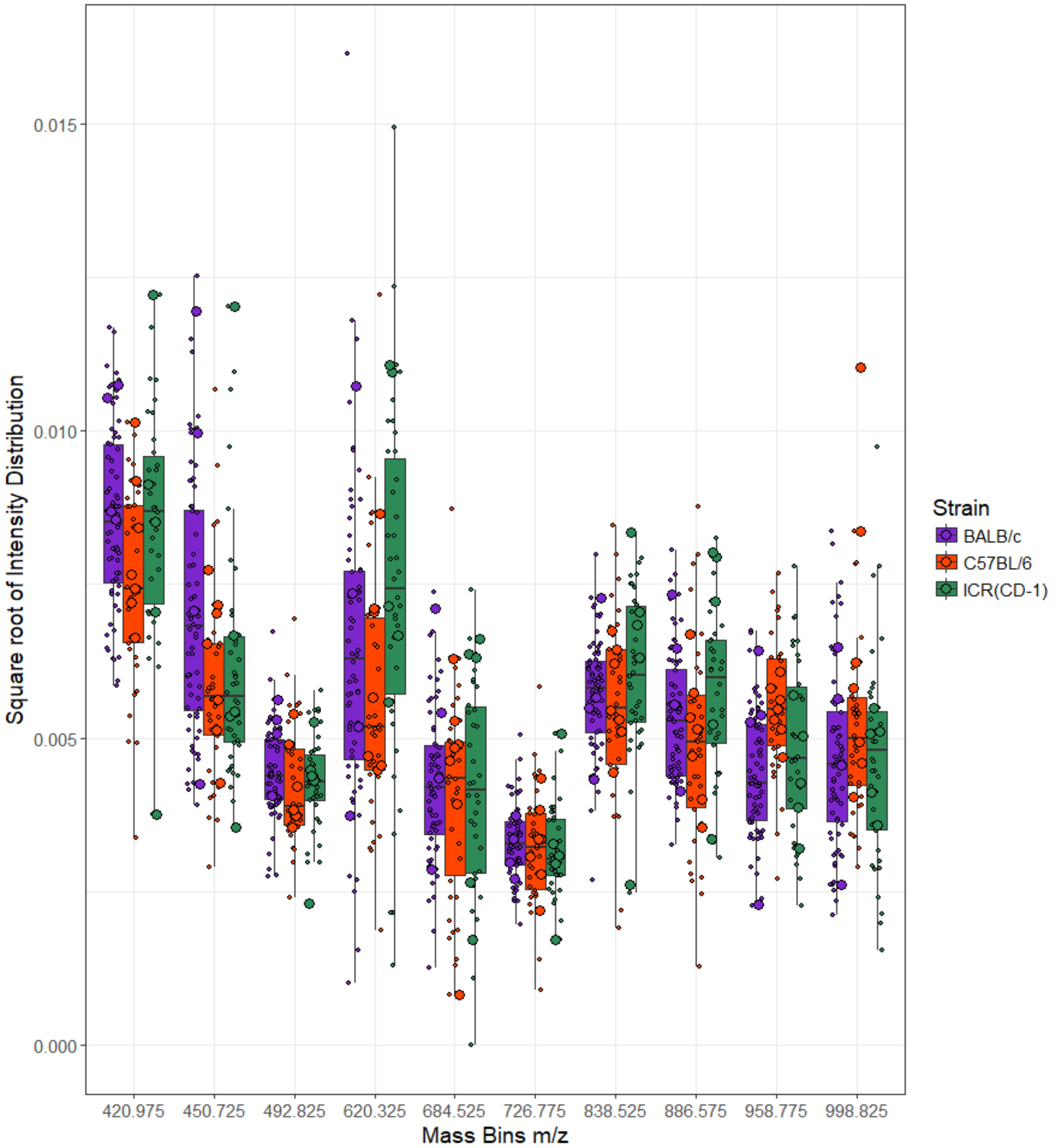

The standard deviation (sd) based on ten random forest classification runs, comparing true classification with randomisation of samples. Random forest accuracy (Rf) is 100 minus ‘out of bag’ error and prediction accuracy (Pa) the % of test samples correctly assigned. No split: The data was not split into training and test sets and the random forest model was created using 100% of the data (whole data set)

Factor	Rf	sd	Range	Pa	sd	Range
Sex	78%	2.71	73-82%	78%	5.31	71-85%
Sex – No split (whole data set)	78%	0.61	77-79%			
Age	84%	1.81	81-87%	84%	3.83	79-90%
Age – No split (whole data set)	84%	0.91	82-85%			
Age – Under sampling	79%	4.67	71-88%	79%	7.59	70-90%
Age – Under sampling, No split	77%	3.18	72-81%			
Strain	83%	2.78	79-88%	83%	6.21	69-92%
Strain – No split (whole data set)	87%	0.67	86-88%			
Random – Sex ratio	52%	3.94	47-58%	48%	4.40	39-53%
Random - Age ratio	71%	3.42	65-77%	73%	5.92	65-86%
Random – Age, under sampling	41%	7.41	29-52%	43%	3.48	38-48%
Random - Strain ratio	41%	3.40	36-48%	43%	4.28	38-48%

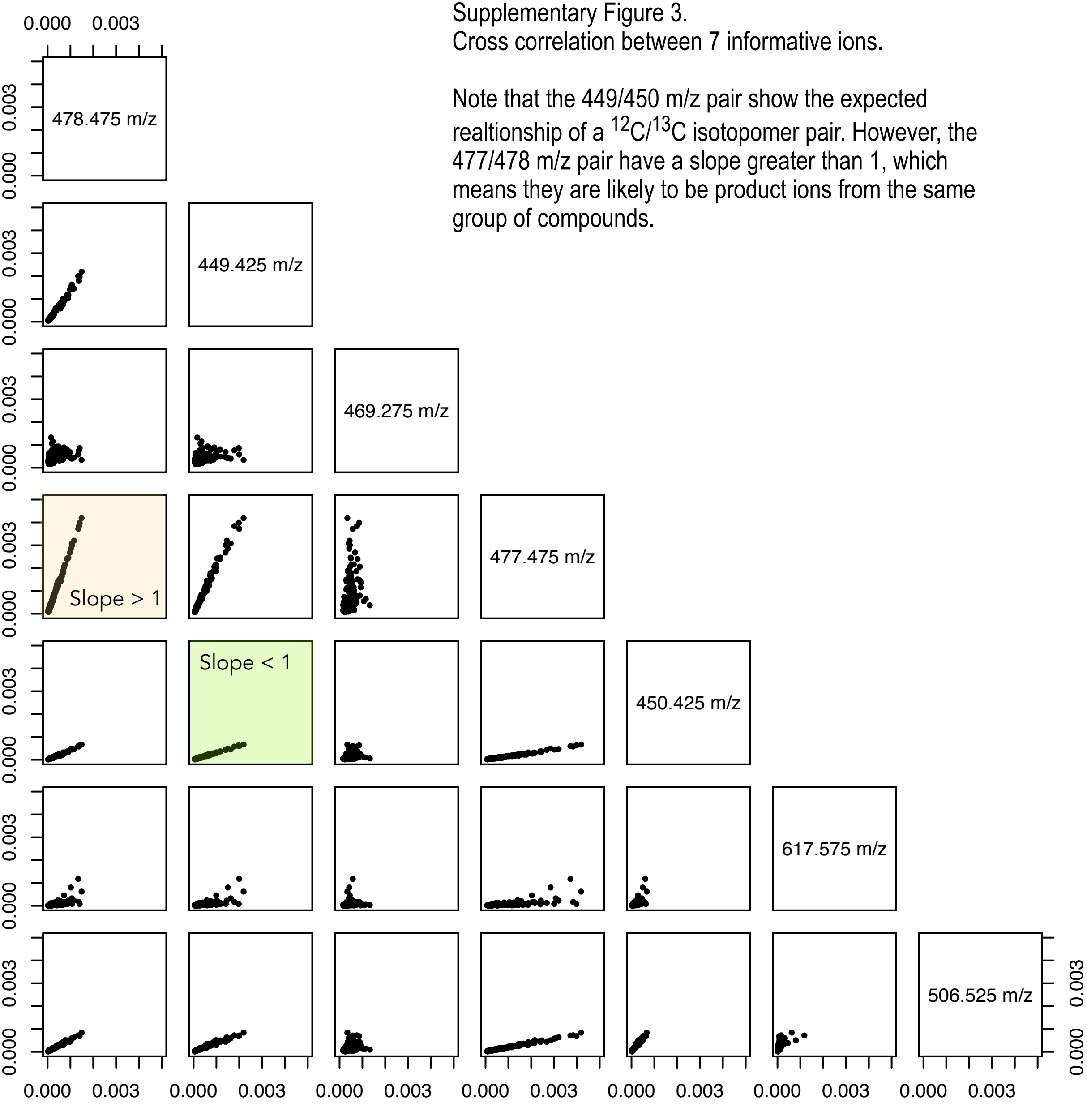

Supplementary Figure 3.
Cross correlation between 7 informative ions.

Note that the 449/450 m/z pair show the expected realtionship of a $^{12}C/^{13}C$ isotopomer pair. However, the 477/478 m/z pair have a slope greater than 1, which means they are likely to be product ions from the same group of compounds.

Supplementary Table 1. The number of individuals for each sex of every species from bred in the laboratory (lab) or caught in the field (wild)

Species	Lab or Wild	Sex	No of Individuals
Bank vole	Lab	Female	10
		Male	10
	Wild	Female	35
		Male	42
		Unknown	3
Field Vole	Lab	Female	10
		Male	8
	Wild	Female	10
		Male	27
		Unknown	3
House Mouse	Lab	Female	11
		Male	10
	Wild	Female	0
		Male	48
Rat	Lab	Female	10
		Male	10
	Wild	Unknown	29
Wood Mouse	Lab	Female	10
		Male	6
	Wild	Female	30
		Male	43
		Unknown	2

The number of individuals of each strain, age and sex used in these studies

Sex	Age	Strain	No of Individuals
Female	Adult	BALB.K	12
		BALB/c	36
		C57BL/6	13
		ICR(CD-1)	11
Female	Juvenile	BALB.K	2
		BALB/c	6
		C57BL/6	2
		ICR(CD-1)	3
Male	Adult	BALB.K	12
		BALB/c	18
		C57BL/6	14
		ICR(CD-1)	16
Male	Juvenile	BALB.K	6
		BALB/c	8
		C57BL/6	9
		ICR(CD-1)	8

The number of individuals for each species bred in the laboratory (lab) or caught in the field (wild)

Species	Lab or Wild	No of Individuals
Bank vole	Lab	20
	Wild	80
Field Vole	Lab	18
	Wild	40
House Mouse	Lab	21
	Wild	48
Rat	Lab	20
	Wild	29
Wood Mouse	Lab	16
	Wild	75
