# Supplementary material for: Rapid identification of species, sex and maturity by mass spectrometric analysis of animal faeces
Source: BMC Biol. 2019 Aug 14;17:66. doi: 10.1186/s12915-019-0686-9 (PMC6693146; doi:10.1186/s12915-019-0686-9)
Supplement: Supplementary file 3 — Figure S2. Randomisation tests based on random assignment to classes. (PDF 326 kb) [file 12915_2019_686_MOESM3_ESM.pdf]

Supplementary Figure 2: Randomisation tests

# Number of Individuals Used For The Randomised Classifications

**Species determination.** The lab and wild species data set was randomised using a random number generator to the same ratio as the five different species in the original data

| Classification | Number of Lab Individuals | Number of Wild Individuals |
|---|---|---|
| A | 20 | 79 |
| B | 17 | 41 |
| C | 21 | 5 |
| D | 20 | 28 |
| E | 17 | 138 |

**Sex, age and strain.** The samples for sex, age and strain were randomised using an online random number generator so each random class had the same number of individuals as the original data

| Classificatio n | Factor | Number of Individuals | % of total individuals |
|---|---|---|---|
| Sex | Random One | 85 | 48 |
| | Random Two | 91 | 52 |
| Age | Random One | 132 | 75 |
| | Random Two | 44 | 25 |
| Strain | Random One | 38 | 26 |
| | Random Two | 38 | 47 |
| | Random Three | 68 | 26 |

**Randomisation test: Entire Data Set**

A random 50% of all samples including all sex, age and strain types assigned as class ‘Random One’ the other half as class ‘Random Two’ using an online random number generator. These randomly assigned classes were then used as input for random forest and LDA.

All Data

| Predicted Classification | Random 1 n=88 | Random 2 n=88 |
|---|---|---|
| Random 2 | 50% | 44% |
| Random 1 | 50% | 56% |

True Classification

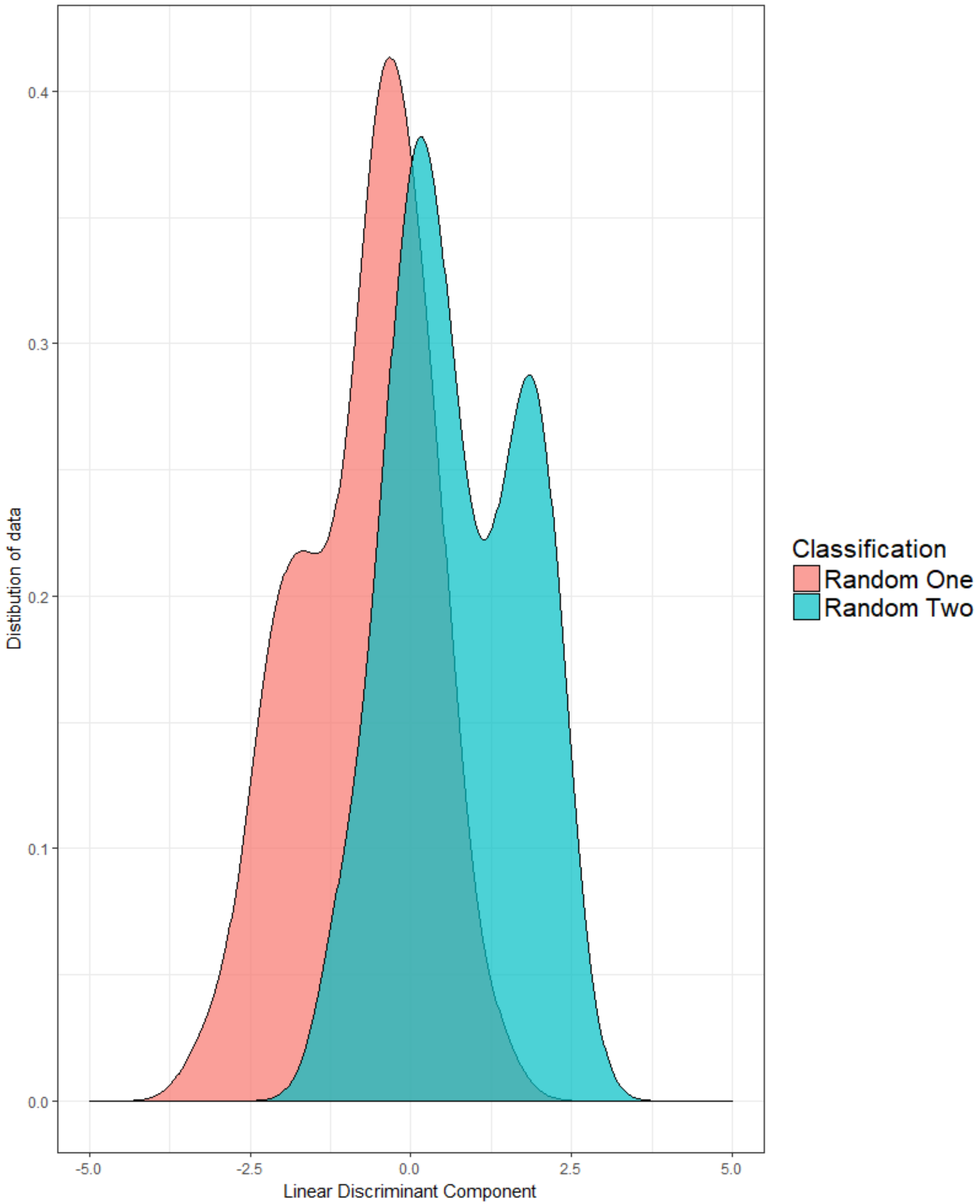

**Randomisation test: sex**

Randomised using the same ratio as Male and Female

All Data

| Predicted Classification | Random 1 (n=85) | Random 2 (n=91) |
|---|---|---|
| Random 2 | 48% | 51% |
| Random 1 | 52% | 49% |

True Classification

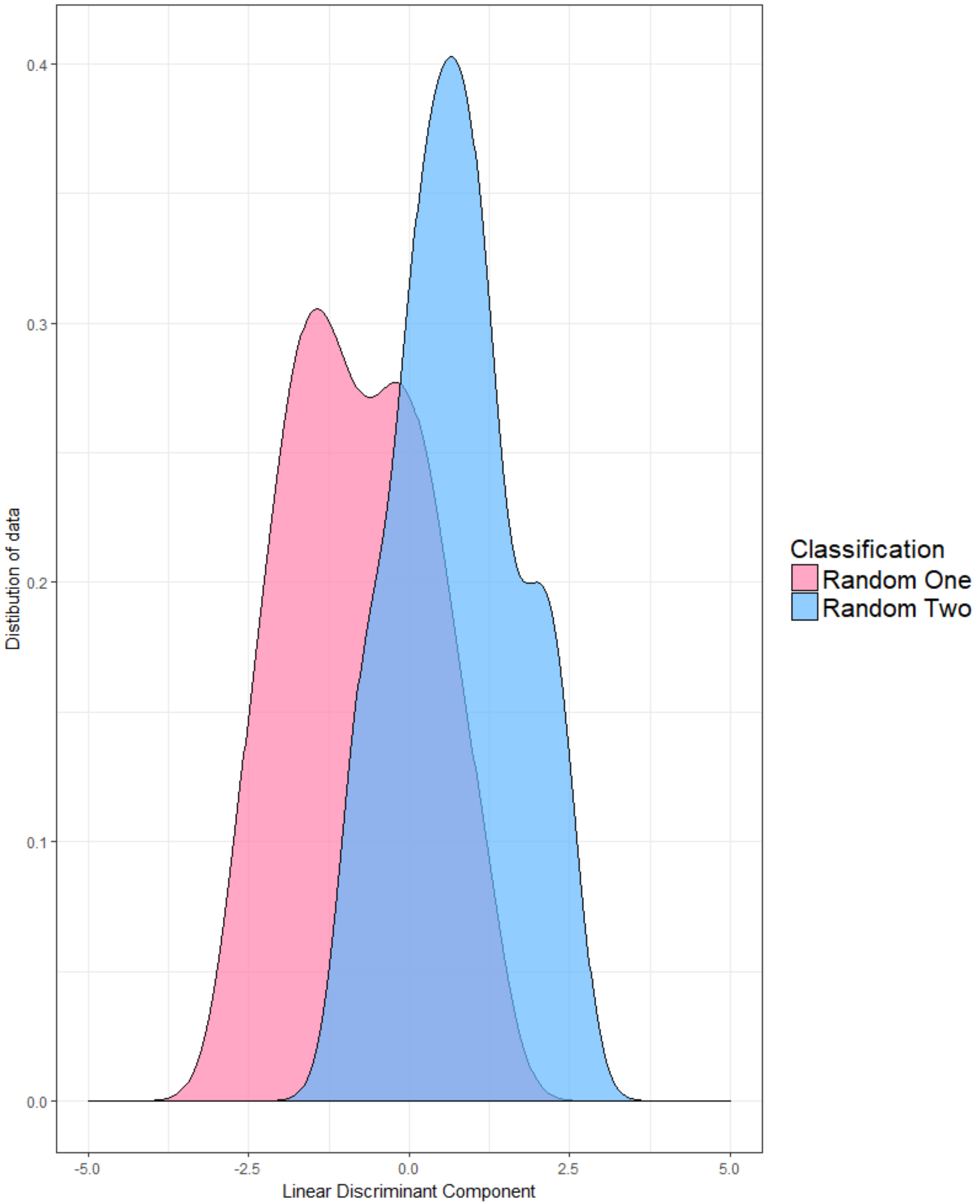

**Randomisation test: age**

Same ratio as Adults and Juveniles

All Data

| Predicted Classification | Random 1 (n=132) | Random 2 (n=44) |
|---|---|---|
| Random 2 | 4% | 5% |
| Random 1 | 96% | 95% |

Under-sampling

| Predicted Classification | Random 1 (n=44) | Random 2 (n=44) |
|---|---|---|
| Random 2 | 61% | 48% |
| Random 1 | 39% | 52% |

True Classification

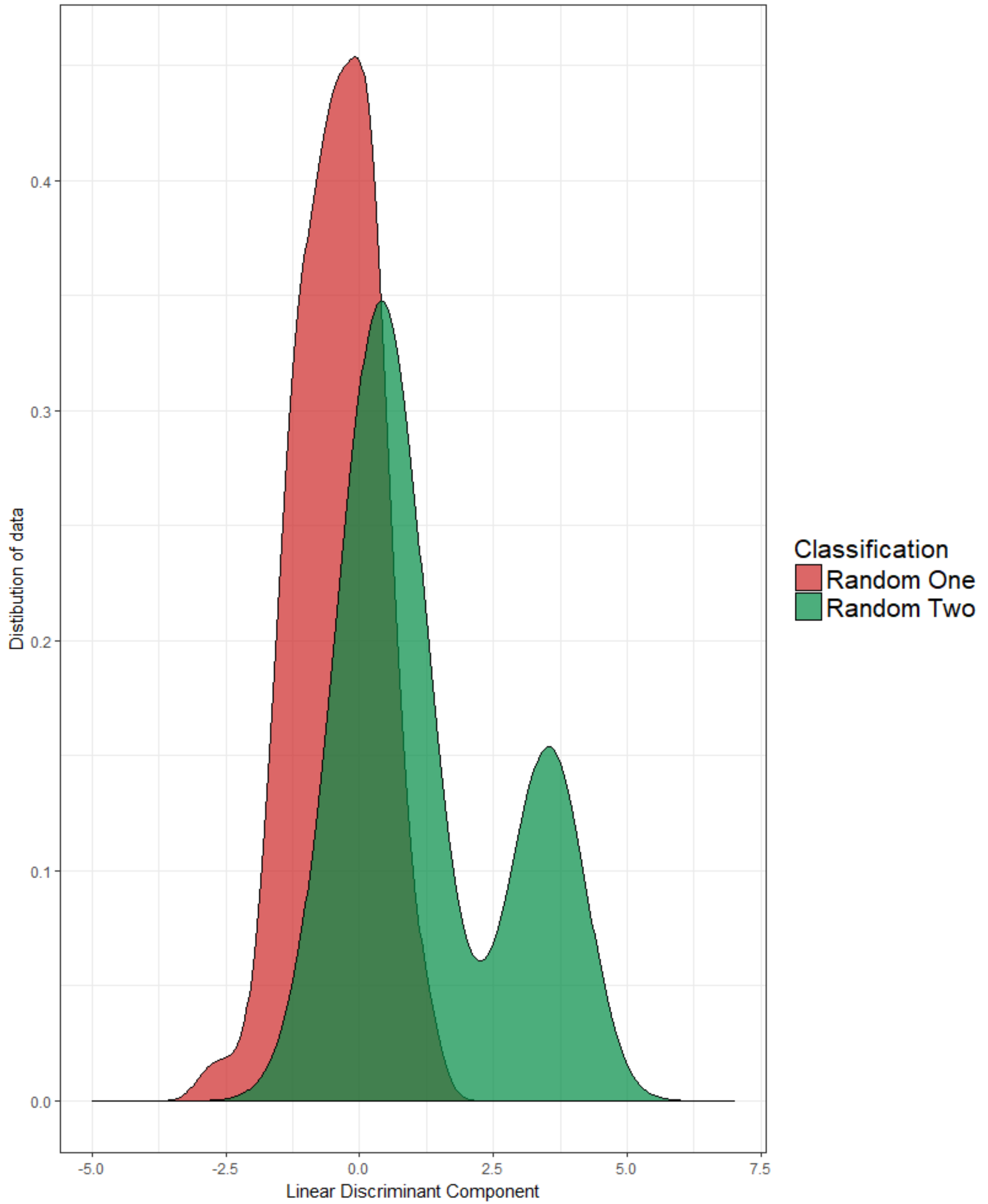

**Randomisation test: sex**

Ten random m/z bin were selected using a random number generator and the intensity distributions are displayed. Correct classifications: small symbols, incorrect classification: large symbols.

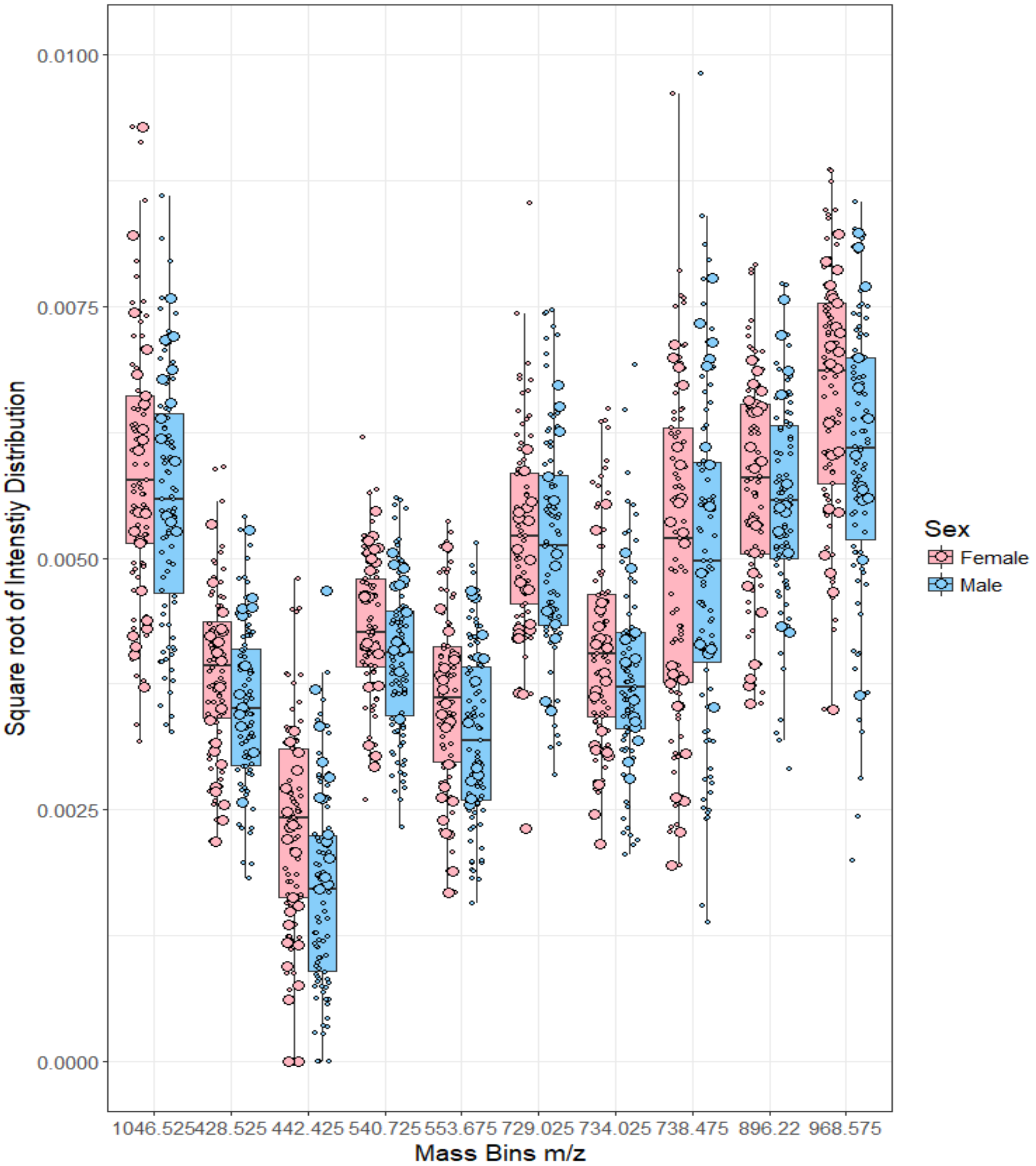

**Randomisation test: age**

Ten random m/z bins were selected using a random number generator and the intensity distributions are displayed. Correct classifications: small symbols, incorrect classification: large symbols.

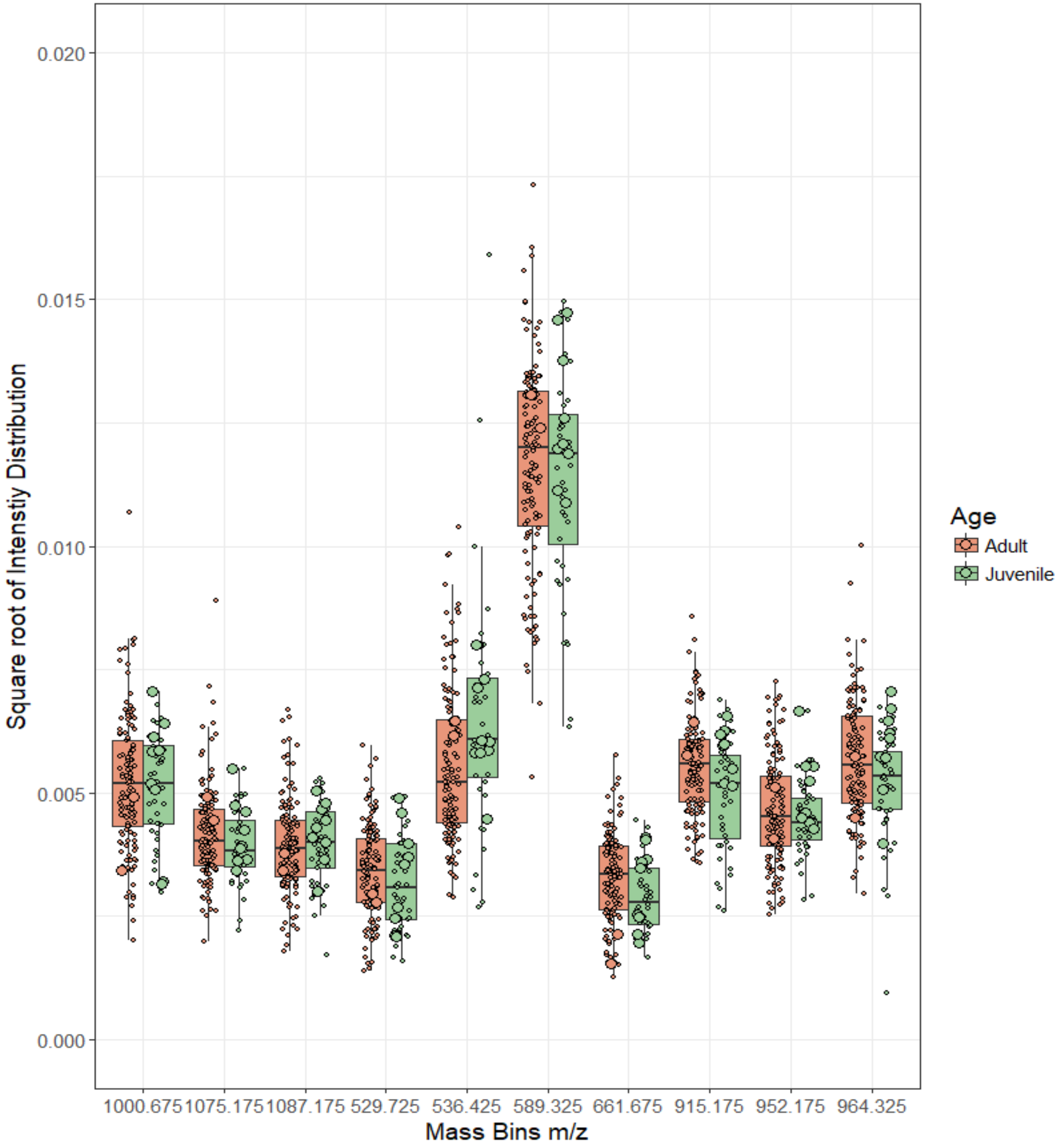

**Randomisation test: strain**

Samples from the three strains were randomised using the same ratio as the source samples.

### All Strain Data

| | Random 1 n=38 | Random 2 n= 38 | Random 3 n= 69 |
|---|---|---|---|
| Random 3 | 86% | 88% | 81% |
| Random 2 | 6% | 0% | 7% |
| Random 1 | 7% | 12% | 12% |

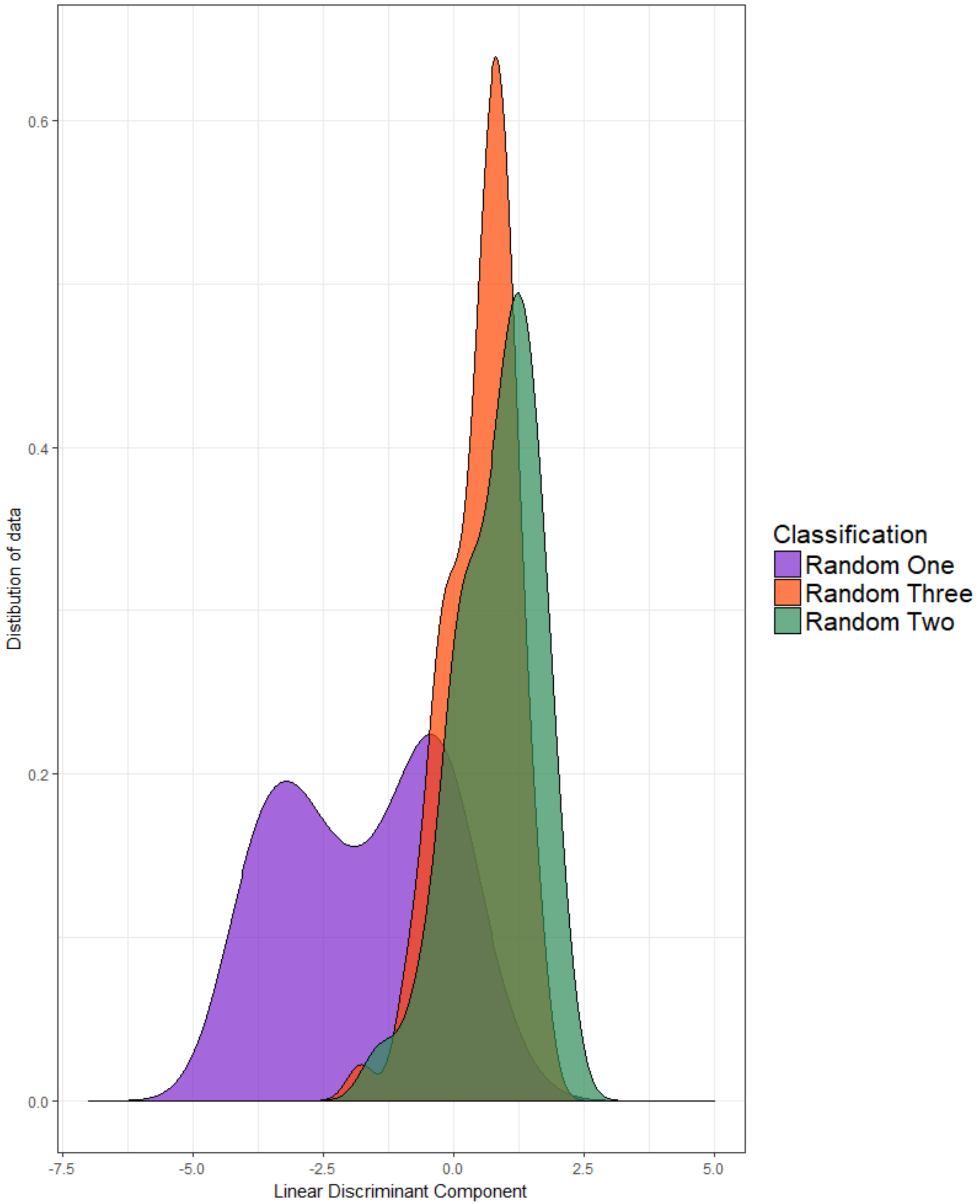

**Randomisation test: strain**

Ten random m/z bin were selected using a random number generator and the intensity distributions are displayed. Correct classifications: small symbols, incorrect classification: large symbols.

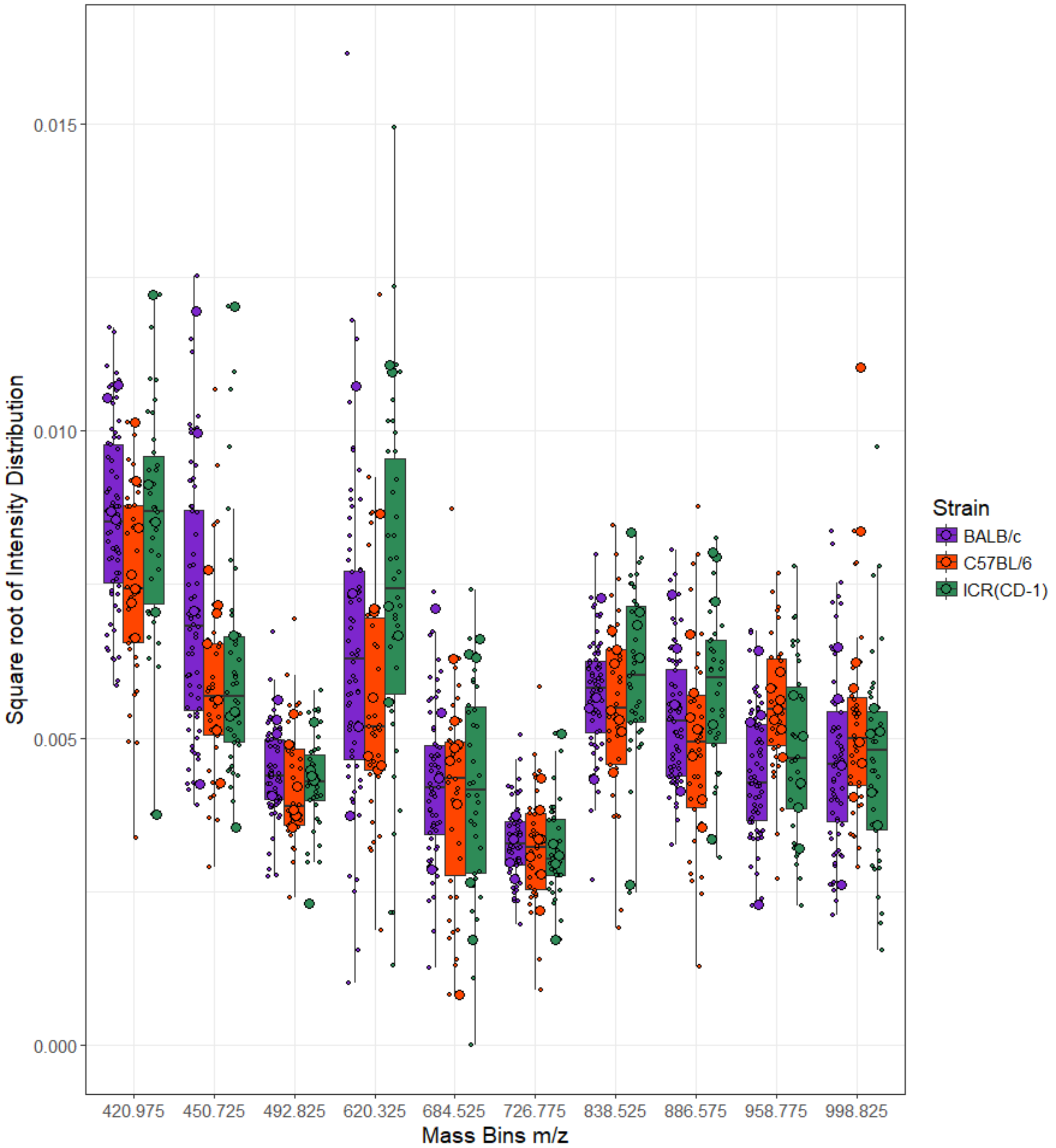

The standard deviation (sd) based on ten random forest classification runs, comparing true classification with randomisation of samples. Random forest accuracy (Rf) is 100 minus ‘out of bag’ error and prediction accuracy (Pa) the % of test samples correctly assigned. No split: The data was not split into training and test sets and the random forest model was created using 100% of the data (whole data set)

| Factor | Rf | sd | Range | Pa | sd | Range |
|---|---|---|---|---|---|---|
| Sex | 78% | 2.71 | 73-82% | 78% | 5.31 | 71-85% |
| Sex – No split (whole data set) | 78% | 0.61 | 77-79% | | | |
| Age | 84% | 1.81 | 81-87% | 84% | 3.83 | 79-90% |
| Age – No split (whole data set) | 84% | 0.91 | 82-85% | | | |
| Age – Under sampling | 79% | 4.67 | 71-88% | 79% | 7.59 | 70-90% |
| Age – Under sampling, No split | 77% | 3.18 | 72-81% | | | |
| Strain | 83% | 2.78 | 79-88% | 83% | 6.21 | 69-92% |
| Strain – No split (whole data set) | 87% | 0.67 | 86-88% | | | |
| Random – Sex ratio | 52% | 3.94 | 47-58% | 48% | 4.40 | 39-53% |
| Random - Age ratio | 71% | 3.42 | 65-77% | 73% | 5.92 | 65-86% |
| Random – Age, under sampling | 41% | 7.41 | 29-52% | 43% | 3.48 | 38-48% |
| Random - Strain ratio | 41% | 3.40 | 36-48% | 43% | 4.28 | 38-48% |
