# Supplementary material for: Rapid identification of species, sex and maturity by mass spectrometric analysis of animal faeces
Source: BMC Biol. 2019 Aug 14;17:66. doi: 10.1186/s12915-019-0686-9 (PMC6693146; doi:10.1186/s12915-019-0686-9)
Supplement: Supplementary file 4 — Figure S3. Ion intensity cross-correlation analysis. (PDF 65 kb) [file 12915_2019_686_MOESM4_ESM.pdf]

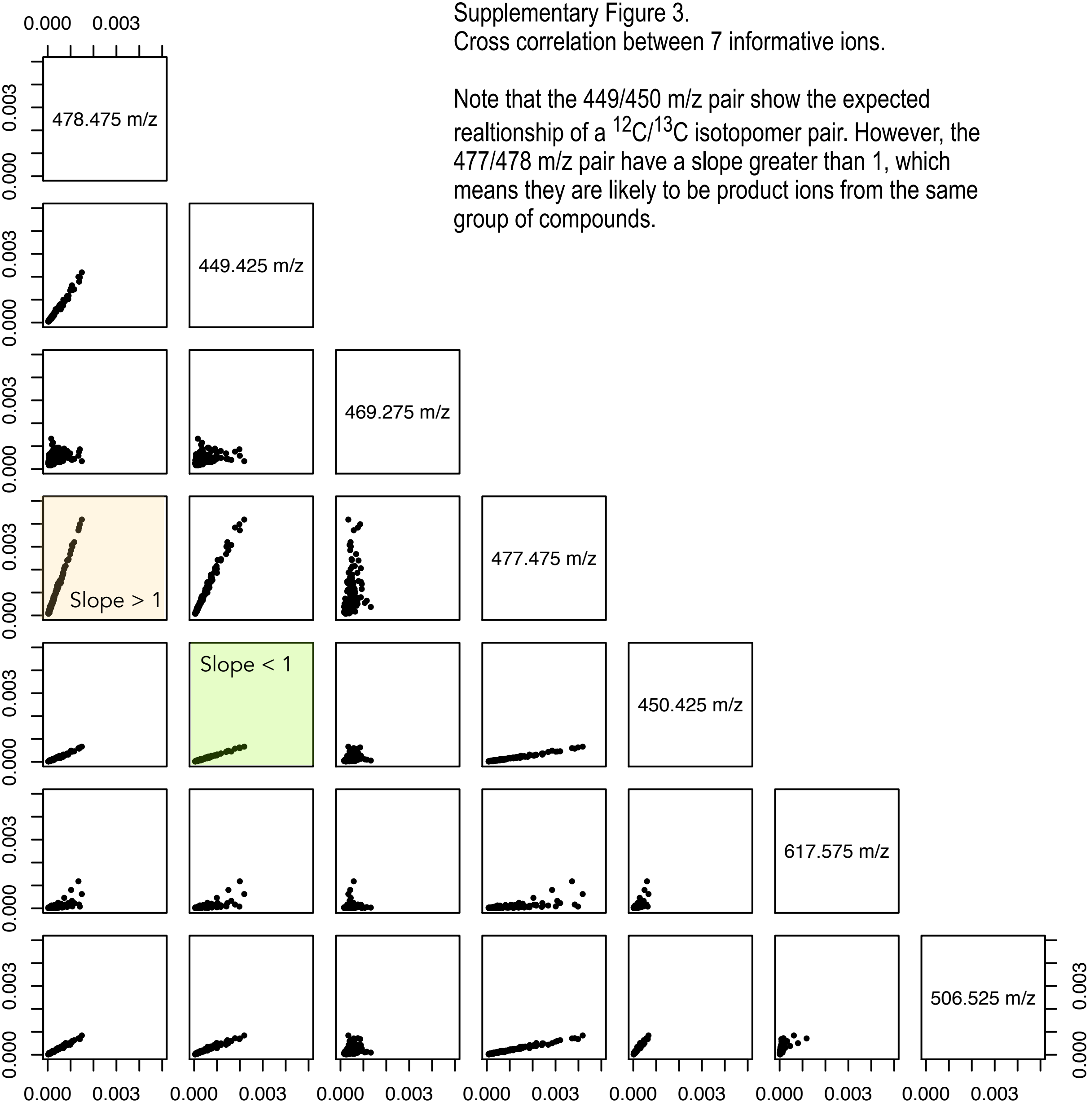

Supplementary Figure 3.
Cross correlation between 7 informative ions.

Note that the 449/450 m/z pair show the expected realtionship of a $^{12}C/^{13}C$ isotopomer pair. However, the 477/478 m/z pair have a slope greater than 1, which means they are likely to be product ions from the same group of compounds.
