# Supplementary material for: Rapid identification of species, sex and maturity by mass spectrometric analysis of animal faeces
Source: BMC Biol. 2019 Aug 14;17:66. doi: 10.1186/s12915-019-0686-9 (PMC6693146; doi:10.1186/s12915-019-0686-9)

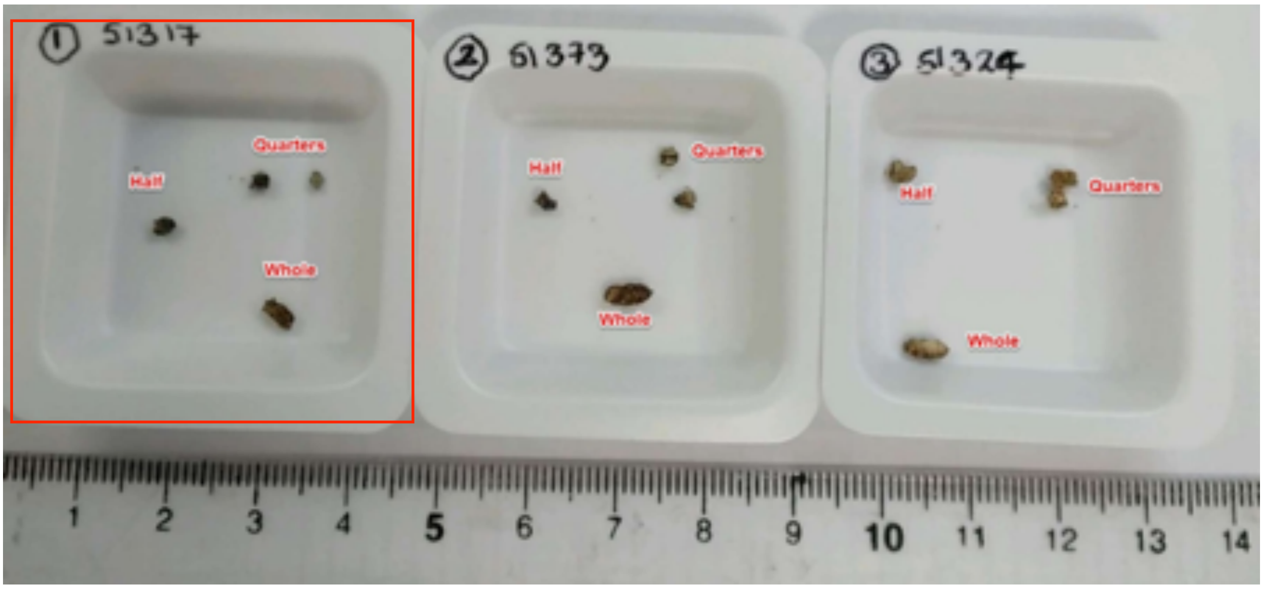

Faecal pellets 51317

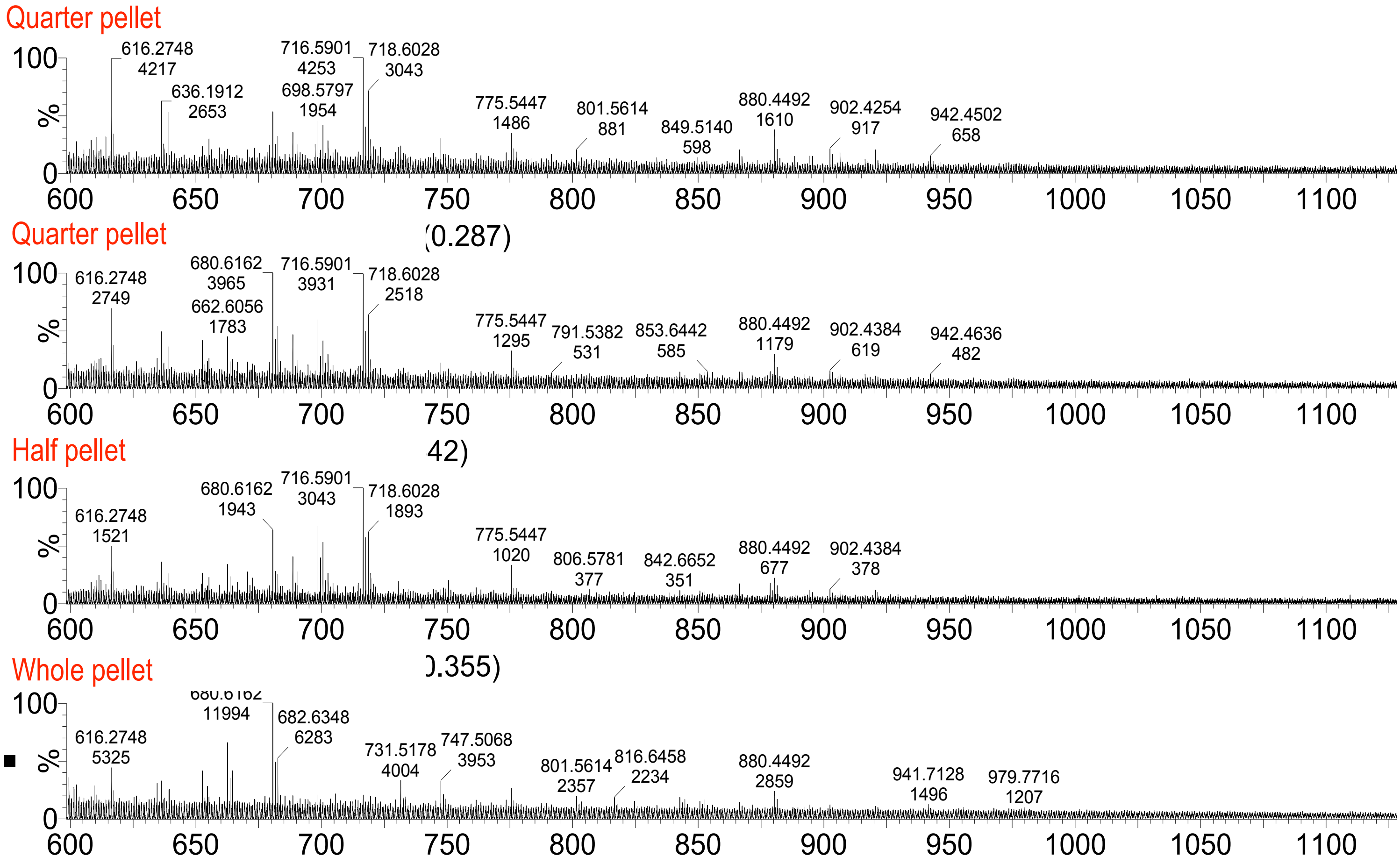

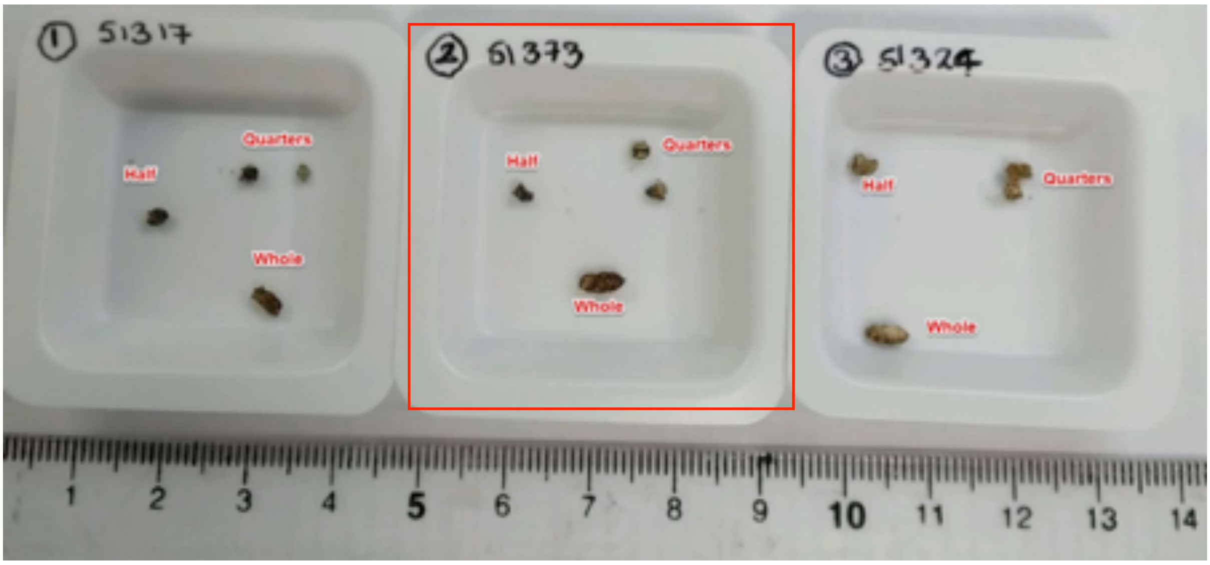

Faecal pellets 51373

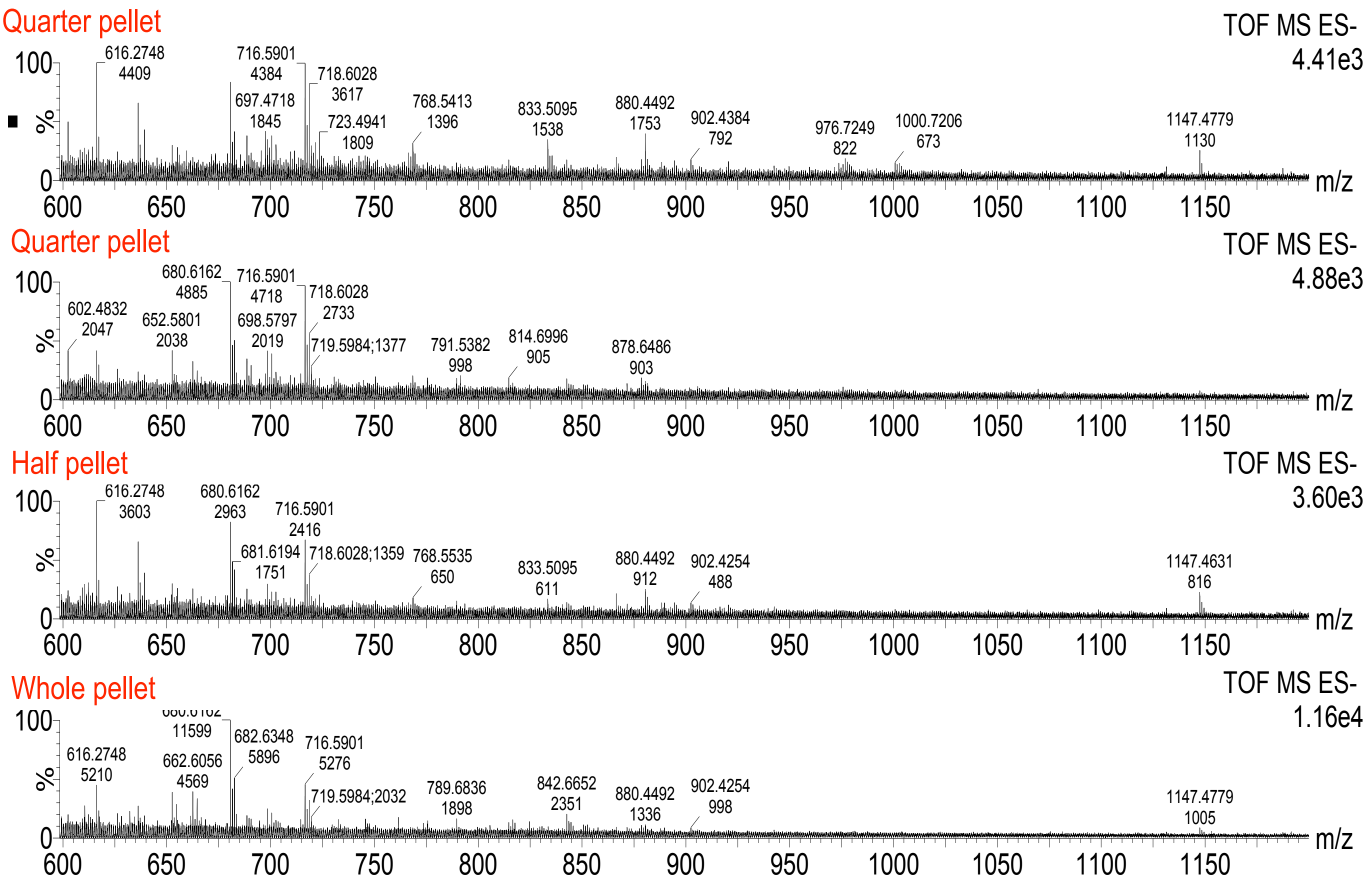

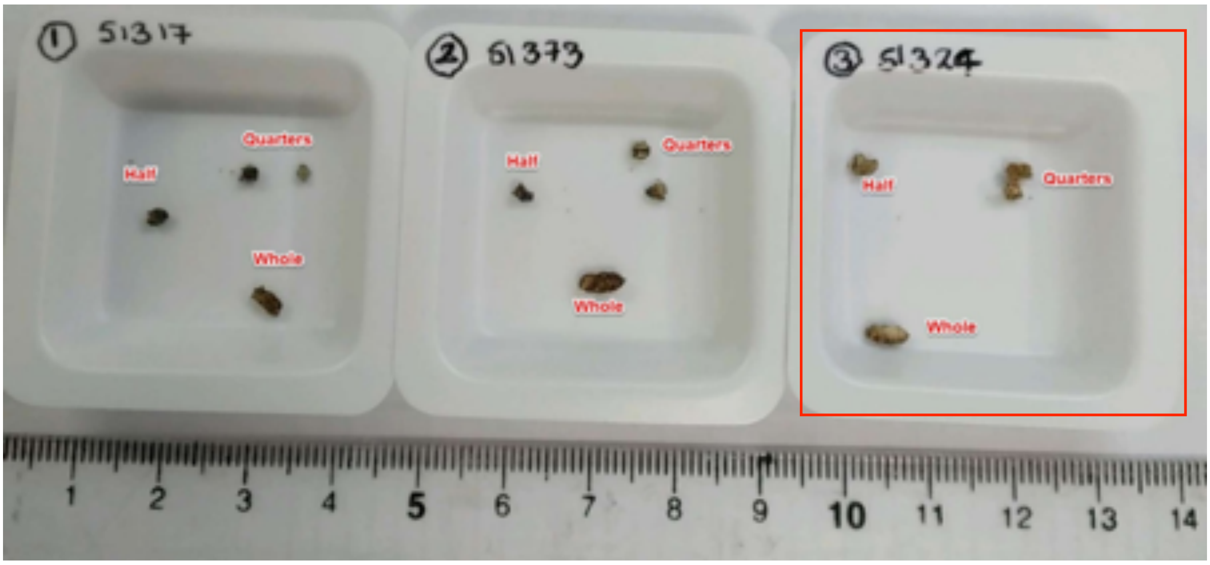

Faecal pellets 51324

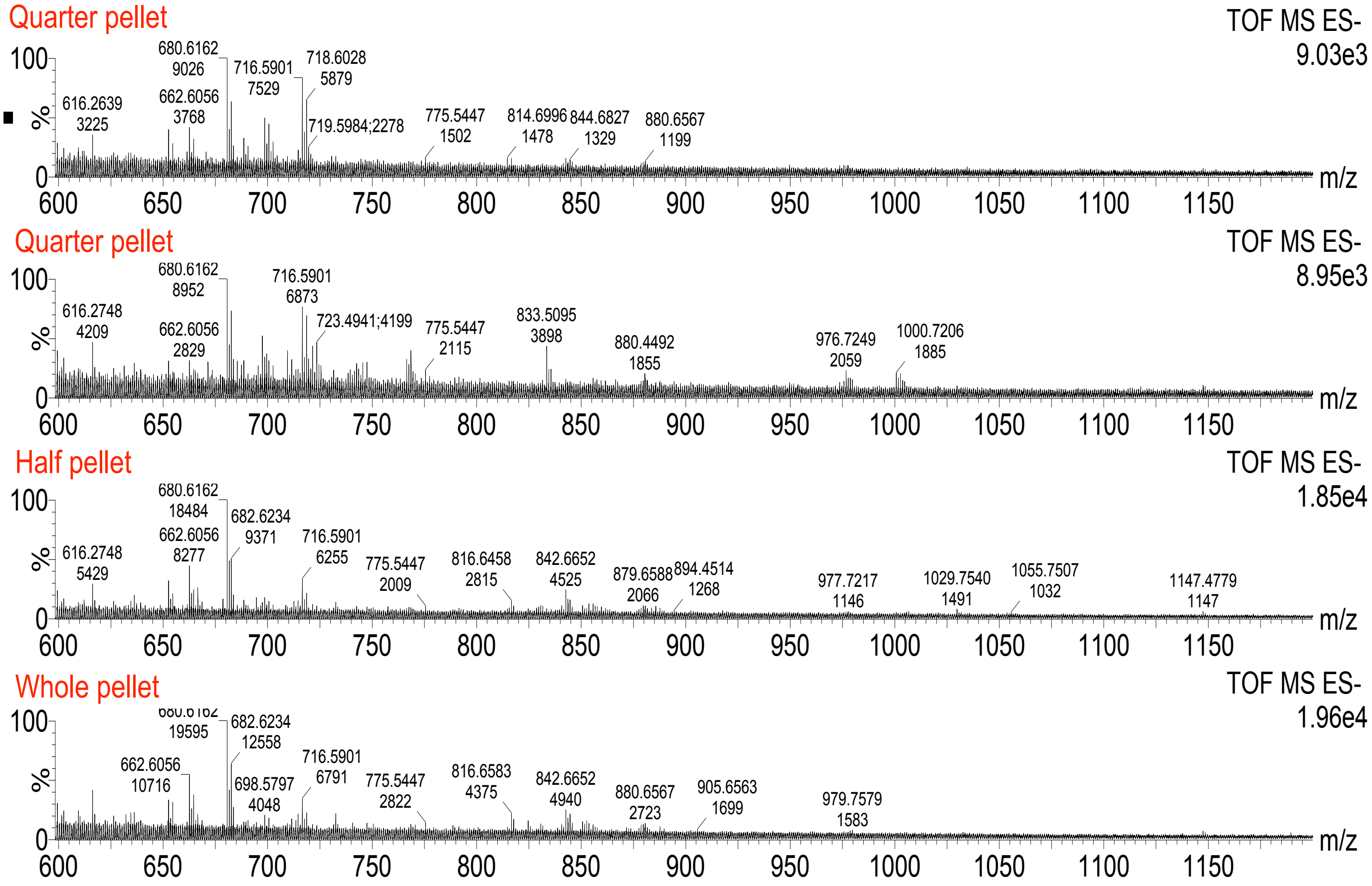

Supplement: Supplementary file 5 — Figure S4. Demonstration of REIMS spectra derived from one half, or one quarter, of mouse faecal pellets. (PDF 2071 kb) [file 12915_2019_686_MOESM5_ESM.pdf]
