# Supplementary material for: Rapid identification of species, sex and maturity by mass spectrometric analysis of animal faeces
Source: BMC Biol. 2019 Aug 14;17:66. doi: 10.1186/s12915-019-0686-9 (PMC6693146; doi:10.1186/s12915-019-0686-9)
Supplement: Supplementary file 6 — Table S1. Numbers of donors of faecal samples used in this study. (PDF 46 kb) [file 12915_2019_686_MOESM6_ESM.pdf]

Supplementary Table 1. The number of individuals for each sex of every species from bred in the laboratory (lab) or caught in the field (wild)

Species	Lab or Wild	Sex	No of Individuals
Bank vole	Lab	Female	10
		Male	10
	Wild	Female	35
		Male	42
		Unknown	3
Field Vole	Lab	Female	10
		Male	8
	Wild	Female	10
		Male	27
		Unknown	3
House Mouse	Lab	Female	11
		Male	10
	Wild	Female	0
		Male	48
Rat	Lab	Female	10
		Male	10
	Wild	Unknown	29
Wood Mouse	Lab	Female	10
		Male	6
	Wild	Female	30
		Male	43
		Unknown	2

The number of individuals of each strain, age and sex used in these studies

Sex	Age	Strain	No of Individuals
Female	Adult	BALB.K	12
		BALB/c	36
		C57BL/6	13
		ICR(CD-1)	11
Female	Juvenile	BALB.K	2
		BALB/c	6
		C57BL/6	2
		ICR(CD-1)	3
Male	Adult	BALB.K	12
		BALB/c	18
		C57BL/6	14
		ICR(CD-1)	16
Male	Juvenile	BALB.K	6
		BALB/c	8
		C57BL/6	9
		ICR(CD-1)	8

The number of individuals for each species bred in the laboratory (lab) or caught in the field (wild)

Species	Lab or Wild	No of Individuals
Bank vole	Lab	20
	Wild	80
Field Vole	Lab	18
	Wild	40
House Mouse	Lab	21
	Wild	48
Rat	Lab	20
	Wild	29
Wood Mouse	Lab	16
	Wild	75
